# Alpha-2-Macroglobulin in Inflammation, Immunity and Infections

**DOI:** 10.3389/fimmu.2021.803244

**Published:** 2021-12-14

**Authors:** Jennifer Vandooren, Yoshifumi Itoh

**Affiliations:** ^1^ Laboratory of Immunobiology, Department of Microbiology, Immunology and Transplantation, Rega Institute for Medical Research, Katholieke Universiteit (KU) Leuven, Leuven, Belgium; ^2^ Kennedy Institute of Rheumatology, University of Oxford, Oxford, United Kingdom

**Keywords:** alpha-2-macroglobulin, proteolysis, inflammation, immunity, infections, macrophages, neutrophils

## Abstract

Alpha-2-macroglobulin is an extracellular macromolecule mainly known for its role as a broad-spectrum protease inhibitor. By presenting itself as an optimal substrate for endopeptidases of all catalytic types, alpha-2-macroglobulin lures active proteases into its molecular cage and subsequently ‘flags’ their complex for elimination. In addition to its role as a regulator of extracellular proteolysis, alpha-2-macroglobulin also has other functions such as switching proteolysis towards small substrates, facilitating cell migration and the binding of cytokines, growth factors and damaged extracellular proteins. These functions appear particularly important in the context of immune-cell function. In this review manuscript, we provide an overview of all functions of alpha-2-macroglobulin and place these in the context of inflammation, immunity and infections.

## Introduction - The Molecular Basis of an Unusual Protease Inhibitor

**Figure 1:**
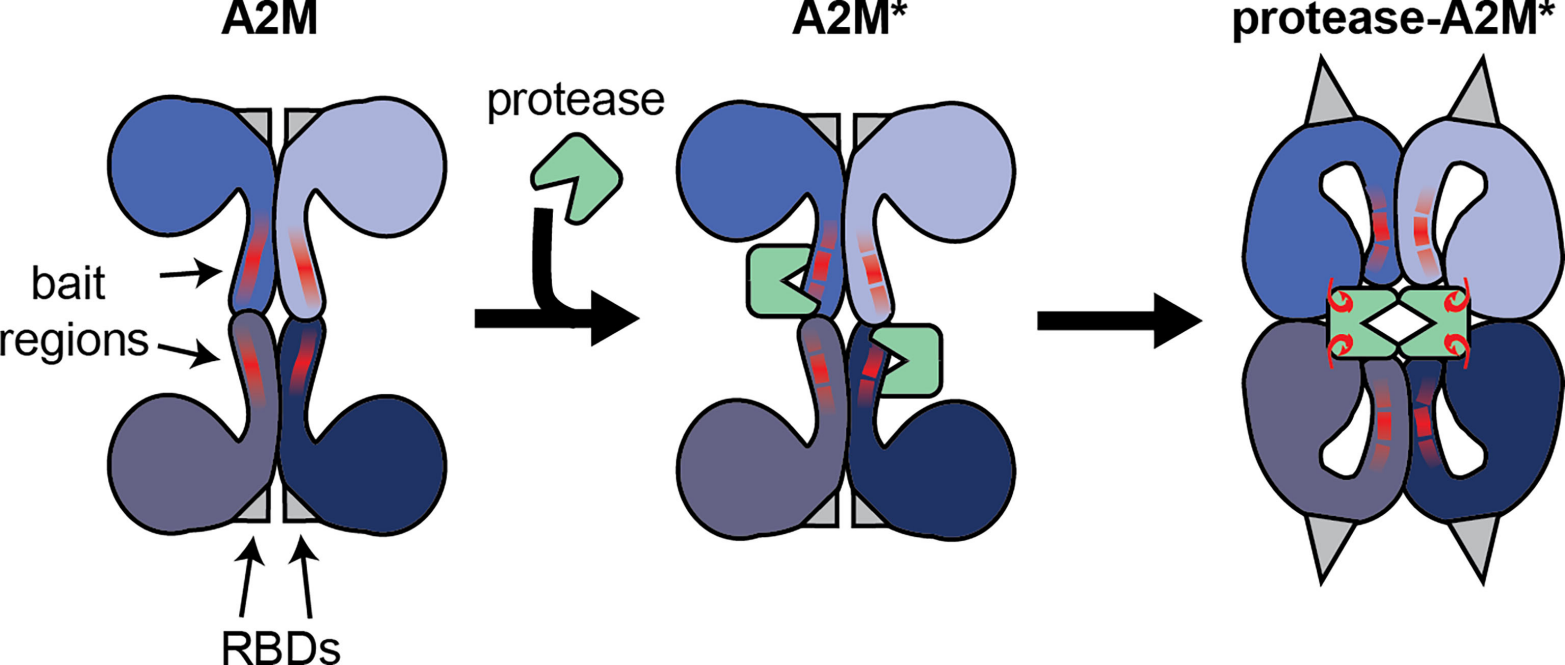
Illustration of the interaction between the A2M tetramer and active endopeptidases. Functional A2M is formed by a non-covalent interaction between two covalently linked dimers. Each monomer contains a protease bait region (red lines) and a buried receptor binding domain (RBDs, gray triangles). Active proteases can cleave the bait region, resulting in A2M activation (A2M*) and conformational rearrangement. This process results in physical trapping of the protease and the exposure of a reactive thioester bond which may result in the formation of a covalent bond between A2M and protease lysine residues (red hooks). Simultaneously, the receptor binding domains are exposed to the protein surface, thereby enabling A2M* to bind its cell-surface receptors.

Alpha-2-macroglobulin (A2M) is a member of MEROPS clan I39 which has seven members in humans and two in mice. All of these proteins can interact with a broad range of endopeptidases. A2M in particular is considered an inhibitor of active endopeptidases of all catalytic types (1). This broad inhibition range stems from its unique mechanism of action. Whereas most protease inhibitors directly interfere with the protease active site, the inhibitory mechanism of A2M works through the formation of a tetrameric cage around active proteases, thereby physically obstructing the interaction between proteases and substrates. This mechanism is sometimes referred to as the protease ‘snap-trap’ or ‘venus-flytrap’ mechanism (2). As a consequence, proteases ‘trapped’ by A2M are prevented from cleaving large substrate molecules (e.g. collagen), while the digestion of small peptides (sneaking into the A2M cage) remains intact (3). The ability of A2M to selectively capture only active proteases, relies on the presence of a ‘bait region’, which is a stretch of amino acids functioning as an exceptionally good substrate for endopeptidases of all catalytic types (4–6). Upon proteolytic cleavage of the bait region by a protease, A2M becomes ‘activated’ (A2M*) and undergoes a conformational change, thereby ‘trapping’ active proteases within its tetrameric cage (>720 kDa) (**Figure 1**). In addition to sterically capturing proteases, A2M* also exposes a reactive thioester which interacts with small primary amines in the protease to form covalent A2M/protease complexes (protease-A2M*) (7, 8). *In vitro*, small nucleophiles such as methylamine (MA) and other low-molecular-weight primary amines are used to cause a conformational rearrangement of the tetramer that is likely similar to the peptidase induced form (9). We will abbreviate this form as A2M**.

Another essential feature of A2M relies on the presence of the receptor binding domains (RBDs, **Figure 1**) that allow A2M to bind specific cell surface receptors. In native A2M, the RBDs are buried within the protein. However, during the conformational rearrangements associated with the transition into A2M*, the RBDs are exposed onto the surface of the protease-A2M* complex. Consequently, this ‘flags’ protease-A2M* complexes for uptake by cells through low density lipoprotein receptor–related protein-1 (LRP-1) (10). In human blood, binding of protease-A2M* complexes to LRP-1 is thought to result in rapid clearance by the liver and provides another level of protease regulation by A2M (11). In addition, this domain is also thought to bind cell surface glucose-regulated protein (GRP)78, also called binding immunoglobulin protein (BiP) or heat shock 70 kDa protein 5 (HSPA5) (12, 13), thereby triggering several cell signaling pathways (*see chapter on the endocytic and signaling receptors*).

Since the first discoveries of A2M as a moderator of proteolysis, broader functions for A2M have been proposed, including the enhancement of immune and cancer cell migration and proliferation (13, 14), the promotion of antigen uptake, processing and presentation by antigen presenting cells (15, 16), the ability of A2M to function as a carrier molecule for cytokines and growth factors (17, 18) and the removal of damaged extracellular proteins (19). In this review manuscript, we place A2M in the context of the immune system and defense against invading microorganisms. We discuss the significance of A2M in neutrophil, monocyte/macrophage and lymphocyte biology, and its relevance in inflammatory diseases and infections.

## A2M and the Complement System

**Figure 2:**
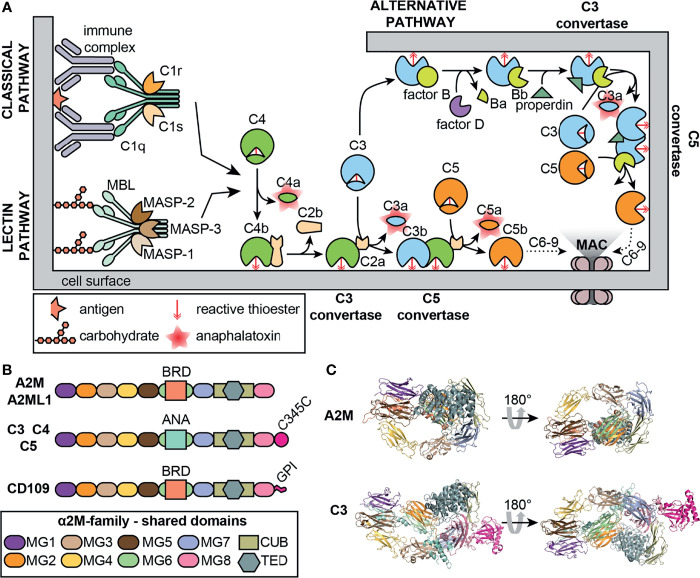
The complement system and A2M. **(A)** Overview of the classical, lectin and alternative complement pathways. MAC, membrane attack complex; MASP, mannose-binding protein-associated serine protease; MBL, mannose-binding lectin. **(B)** Overview of the domain organization of a selection of human proteins belonging to the A2M family (MEROPS protease inhibitor family I39). A2ML1, A2M-like-1; ANA, anaphylatoxin domain; BRD, bait-region domain; C345C, C-terminal extension of C3-5; CUB, complement C1r/C1s, Uegf, Bmp1; GPI, glycosyl-phosphatidyl-inositol linker; MG, macroglobulin-like domains; TED, thioester domain. **(C)** comparison of the crystal structures of the A2M monomer (PDB structure: 4ACQ) (2) and C3 (PDB structure: 2A73) (20). Domain colors are based on the colors used in Panel **(B)** For A2M, part of the bait region and the MG8/RBD are not shown.

The complement system is a key part of the immune system that comprises an evolutionarily ancient component of the host defense. Central to this system is the cleavage of complement factors (C3 and C4), followed by conformational changes and the exposure of an internal thioester which binds hydroxyl or amino groups of neighboring glycoproteins proteins (**Figure 2A**). This process marks malignant cells for removal (opsonization) or generates new protein complexes dedicated to further activate the complement cascade (21). Several striking parallels between A2M and the complement system exist. Similar to C3 and C4, A2M undergoes proteolysis, a conformational change and exposes an internal thioester which binds and traps active protease (22). Hence, together with C3 and C4, A2M is classified as a member of the thioester-containing proteins (TEPs). Similarly, the A2M family of proteins (A2Ms, MEROPS clan I39) can be separated into two classes; the protease inhibitors and the complement factors (23). In addition, also a membrane associated member of the A2M family (CD109) exists (**Figure 2B**) **(** 24). Interestingly, it is thought that C3 evolved from a gene duplication of A2M (20) and that all A2Ms evolved from a core structure of eight homologous domains (24). Indeed, comparison of the molecular models of A2M and C3 illustrates considerable similarities between both proteins (**Figure 2C**). For a comprehensive overview of the evolutionary origins of A2M and their structure and function the reader is referred to a manuscript by Garcia-Ferrer *et al.* (25).

In *Limulus polyphemus* (the Atlantic horseshoe crab), A2M acts as a protease inhibitor and, once activated, as an inhibitor of the haemolytic mechanism in *Limulus* haemolymph. A2M binds to erythrocyte surfaces and it was speculated whether it could act as an opsonin (22, 26–28). Therefore, it is tempting to wonder if A2M - being an ancestral thioester-containing protein - might have acted as the initial “opsonic” system, activated by proteases derived from invading organisms (e.g. virulence factors) and subsequently binding to the protease-producing organism (1, 29, 30). A main difference, however, between A2M and complement components is that the latter are secreted as monomeric proteins whereas human A2M forms a tetrameric structure, permitting physical entrapment of active proteases (29).

Several direct interactions between human A2M and proteins of the complement system have also been discovered. In human serum, a complex was found between mannose-binding protein (MBP), MBP-associated serine protease (MASP, a protease with complement activation activity in the lectin pathway) and A2M, suggesting a regulatory role for A2M in the lectin pathway of the complement cascade (**Figure 2A**) (31). Furthermore, direct inhibitory activity of A2M on MASPs was shown and others suggested that A2M is mainly an inhibitor of the ancient type protease MASP-1, whereas inhibition of MASP-2 is less efficient (32). An interaction between A2M and MASP-1 was also confirmed by Paréj *et al.*, however, in this study A2M could not abolish lectin pathway activation (33). Also, a direct interaction between MBL and A2M was suggested (34, 35). When screening for human and mouse serum proteins with the capacity to bind to mannan-binding lectin (MBL), A2M was identified. This interaction occurred through the direct binding of MBL carbohydrate recognition domains to oligomannose glycans Man_5–7_ present at Asn^846^ on A2M. This binding site remains accessible both on A2M and A2M*, and binding of MBL to A2M hardly interferes with the ability of MBL to bind mannan-coated surfaces. Interestingly, in the same study C3 and C4 were also identified as MBL-binders. Finally, it was suggested that an ancestral (glycosylated) A2M-like TEP might have generated “arrays” of oligomannose glycans on the surface of microorganisms through inhibition of cell surface proteases. Subsequently, MBL or other lectins could bind to the oligomannose layer, leading to opsonization and activation of the complement system (35). In a recent study, A2M was also identified as the possible antigen causing hexamerization/aggregation of IgG as seen in patients with chronic lymphocytic leukemia and chronic activation of the complement classical pathway. A2M was found to be part of the IgG hexamer complex and present at the cell surface of malignant B lymphocytes through binding with GRP78 (36). The mechanism through which such interaction would occur remains to be determined. In conclusion, several separate studies have provided clues for some involvement of A2M in activation of the complement system. However, the overall relevance of these findings in a biological context or the contribution to pathological processes largely remains to be explored.

## Binding to Cytokines and Growth Factors

As early as the 1970s, researchers discovered that macrophage activation factors and nerve growth factors were bound to A2M in human and mouse serum (37, 38). Next, many more cytokines and growth factors joined the list of A2M-binding proteins (see **Table 1**). Biochemical interaction studies revealed that most cytokines and growth factors preferentially bind protease-activated A2M* or chemically activated A2M**. For example, transforming growth factor (TGF)-β1 (K_D_= 80 ± 11 nM), nerve growth factor (NGF)-β (K_D_= 0.11 ± 0.01 uM), fibroblast growth factor (FGF)-2 (K_D_= 0.59 ± 0.04 uM) and tumor necrosis factor (TNF)-α (K_D_ >0.75 ± 0.10 uM) have increased binding affinity for A2M** and reach their binding equilibrium within 15 minutes. As an exception, TGF-β2 binds both forms equally well (K_D_ ±12 nM) (40, 41, 55, 64).

**Table 1:** A2M interaction with cytokines and growth factors.

Binding partner	Proposed interaction	Biological significance and impact on cytokine/growth factor activity	ref.
**FGF1**	binds to A2M and increasingly to A2M**	–	(39)
**FGF2**	non-covalent contact with A2M**, slowly converts into a covalent interaction	complex forms in human plasma reduced binding to FGF-receptors (BHK-21 cells) reduced ability to stimulate plasminogen activator production (bovine epithelial cells) A2M** inhibits FGF-2–dependent fetal bovine heart endothelial cell proliferation does not affect FGF-2–induced vascular tubule formation on Matrigel or collagen matrix	(39–41)
**FGF-4**	binds A2M, increased binding to A2M**	-	(39)
**FGF-6**	binds A2M, increased binding to A2M**	-	(39)
**IFN-γ**	covalent binding to A2M** and protease-A2M*	no impact on antiproliferative activity (bladder tumor cell line) no influence on induction of MHC class II	(42)
**IL-1β**	covalent binding to A2M** and trypsin-A2M* increased binding to MAC	complex found in human plasma IL-1β/A2M retains IL-1-like activity (mouse thymocytes)	(43–45)
**IL-2**	binds oxidized A2M	–	(46)
**IL-4**	binds A2M and preferentially A2M**	–	(44)
**IL-6**	binds A2M** and MAC better binding to oxidized A2M	complex found in human plasma IL-6 receptor binding intact Stimulation of IL-6-dependent hybridoma cells remains intact protects IL-6 from proteolysis	(44, 46, 47)
**IL-8**	non-covalent binding to A2M**	IL-8/A2M isolated from lungs of ARDS patients no effect on neutrophil chemotaxis protects IL-8 from proteolysis	(48)
**IL-18**	binds mostly to MAC	–	(44)
**NGF-β**	non-covalent with A2M**, slowly converts into a covalent interaction binds less to oxidized A2M binds A2M between AA 614-797	–	(40, 46, 49)
**PDGF**	non-covalent binding to A2M**, slowly converts into a covalent interaction binds less to oxidized A2M 2 x PDGF per A2M all isoforms bind native A2M and A2M** binds to the growth factor binding site	complex found in human plasma retains mitogenic activity not detected by anti-PDGF antisera blocks receptor binding clearance of PDGF-BB/A2M** from mouse plasma through uptake *via* LRP-1 fusion protein containing the A2M binding site blocks binding to PDGF-β receptor (NIH 3T3 cells)	(17, 40, 49–54)
**TGF-β1**	non-covalent contact with A2M**, slowly converts into a covalent interaction binds less to oxidized A2M binds to the growth factor binding site	complex found in human plasma TGF-β/A2M is the ‘latent’ TGF-β form in plasma A2M impedes binding to cell surface receptors Fast clearance of TGF-β1/A2M* and TGF-β1/A2M** complexes from mouse plasma through liver uptake TGF-β and A2M** synergistically promote SMC proliferation (cultured rat aorta cells) protein with A2M binding site neutralizes TGF-β1 activity (endothelial cell proliferation assays)	(38, 51, 55–60)
**TGF-β2**	non-covalent contact with A2M**, slowly converts into a covalent interaction binds equally well A2M and A2M** binds less to oxidized A2M binds A2M between AA 614-797	A2M inhibits binding to cell surface receptors reduces the anti-proliferative activity of TGF-β1 protein containing the A2M binding site neutralizes TGF-β2 activity in endothelial cell proliferation assays (fetal bovine heart cells)	(40, 56)
**TNF-α**	non-covalent contact with A2M**, slowly converts into a covalent interaction binds to plasmin-A2M* and less to native A2M, trypsin-A2M* or thrombin-A2M* Increased binding to MAC and oxidized A2M	TNF-α binding to MAC suppresses inflammation by inhibition of MAPK p38 phosphorylation TNF-α retains cytotoxic effects on fibroblasts (L929 murine fibroblasts) no effect on antiproliferative activity (bladder tumor cell line)	(40, 42, 44, 46, 61)
**VEGF**	covalent binding does not bind at growth factor binding site binds the interior of A2M** and the exterior of native and protease-activated A2M*	reduced binding to VEGF receptor VEGF/A2M** complexes are internalized and degraded by macrophages (LRP-1-mediated) A2M does not impact VEGF-induced cell proliferation or Ca^2+^ increases (HUVECs).	(62, 63)

A2M*, protease-activated A2M; A2M**, A2M activated through reaction with low molecular-weight primary amines; AA, amino acid; ARDS, adult respiratory distress syndrome; FGF, fibroblast growth factor; HUVECs, Human umbilical vein endothelial cells; IFN, interferon; IL, interleukin; MAC, A2M activated for cytokine binding; NGF, nerve growth factor; PDGF, platelet-derived growth factor; SMC, smooth muscle cell; TGF, transforming growth factor; TNF, tumor necrosis factor; VEGF, vascular endothelial growth factor.

Based on competition experiments it was suggested that several binding sites for cytokines and growth factors exist and that these also depend on the A2M conformation. For example, FGF competes with TGF-β, but not platelet-derived growth factor (PDGF), for binding to A2M (41, 55). All PDGF-isoforms (AA, BB & AB) compete for binding to A2M and A2M**. However, they do not compete for native A2M with TGF-β1, TGF-β2, TNF-α, FGF2, interleukin (IL)-1β and IL-6, whereas for A2M** PDGF competes with TGF-β1 and FGF2 (50). Similarly, it was shown that vascular endothelial growth factor (VEGF) does not compete with TGF-β1 or PDGF (62). TGF-β1 competes for the binding of FGF-2 to A2M and A2M** (39). In addition, A2M only binds certain members of the FGF-family, including FGF-1, -2, -4 and -6, but not FGF-5, -7, -9 or -10 (39).

The nature of the interaction between A2M and cytokines/growth factors can be both non-covalent and covalent. Most often, initial complexes between A2M** and cytokines/growth factors are non-covalent and reversible, and are slowly converted into covalent interactions (40) (**Table 1**). Generally three mechanisms for A2M/cytokine/growth factor binding have been proposed; (i) non-covalent interaction through trapping in the A2M molecular cage, (ii) covalent interaction through thiol-disulphide exchange with a free thiol-group exposed upon A2M activation, (iii) covalent binding *via* the active thioester group, exposed upon protease activation of A2M (28). Interestingly, for some growth factors, an A2M-binding region was identified. A 20 kDa protein, comprising the A2M bait region and neighbouring sequences (amino acids 614-797), could interact with TGF-β1, TGF-β2, PDGF-BB and NGF-β (56). This sequence could also neutralize TGF-β1 and TGF-β2 activity in endothelial cell proliferation assays (fetal bovine heart cells) (56) and the binding of PDGF-BB to PDGF receptors on fibroblasts (NIH 3T3 cells) (49). Subsequently the growth factor binding site (for TGF-β1 and PDGF-BB) was narrowed down to a 16-amino acid peptide (WDLVVVNSAGVAEVGV), containing a high proportion of hydrophobic amino acids (51) and within this sequence one glutamic acid residue was shown to be crucial for binding to PDGF-BB (and not TGF-β1) (52).

For many cytokines/growth factors, the functional implications of binding to A2M remain unknown (18). One possibility is that A2M serves as a reservoir for cytokines and growth factors, and increases their half-life (18). For IL-8/A2M** complexes it was shown that IL-8 retained its potential to induce neutrophil chemotaxis and complexation rendered IL-8 less sensitive to proteolysis by neutrophil elastase (NE) (48). However, when analyzing the plasma clearance of TGF-β1, TGF-β1/A2M* and TGF-β1/A2M** complexes administered to mice, the complexes were efficiently cleared from the circulation through liver uptake (t_1/2_ of 4 min), whereas free TGF-β1 was also found in the lungs (58). This difference in clearance could be explained by receptor-mediated uptake of TGF-β1/A2M* complexes through interaction of the RBDs with LRP-1 (58). However, other researchers showed that blocking of the clearance receptor by excess administration of A2M** did not affect the half-life of TGF-β1 and that endogenous TGF-β1 is mainly bound to native A2M (65). Similarly, TNF-α/plasmin-A2M* complexes injected into mice were efficiently cleared and this clearance could be stopped by blocking the A2M-receptor (61). The same was true for PDGF-BB/A2M** complexes (54). These findings indeed suggest a role for A2M in cytokine regulation and the net effect might be context-dependent. For example, under physiological conditions, cytokines and growth factors might bind native A2M and remain in circulation. In contrast, under conditions with high protease activity, proteolysis of A2M and conversion into A2M*, results in rapid clearance of A2M*/cytokine/growth factor complexes. Unfortunately, *in vivo* evidence to support this hypothesis is limited.

Another possible impact of A2M complex formation is the modulation of growth factor/cytokine-receptor interactions and their associated functions. It was reported that TGF-β and A2M** work synergistically to promote proliferation of cultured smooth muscle cells (SMCs) (60). However, in another study, A2M reduced the ability of TGF-β2 to inhibit lung cell proliferation (CCL-63 mink lung cell line), whereas the activity of TGF-β1 remained unaltered (64). In case of TNF-α, the presence of A2M** or plasmin-A2M* did not affect its cytotoxic effects on cultured fibroblasts (mouse L929 cells) (61). FGF-2 incubation with A2M** reduced FGF-2–induced endothelial cell proliferation. In contrast, interaction with A2M** did not affect vascular tubule formation on Matrigel basement membrane matrix or collagen, likely due to exchange of FGF-2 with extracellular matrix components (39). Hence, the inhibitory effect of A2M** on FGF-2 function might be limited to the fluid phase and may serve to restrict FGF activity to sites of angiogenesis, inflammation, and tissue repair.

The preference of some cytokines/growth factors to bind A2M*/A2M** has been exploited by efforts to generate stable conformational intermediates of A2M*/A2M** with optimized cytokine and growth factor binding capacities (66). Human A2M, treated with a cross-linking reagent to lock the A2M conformation and subsequently with methylamine, bound TGF-β1 and TGF-β2 with higher affinity than other A2M forms. This form successfully inhibited TGF-β1–mediated inhibition of endothelial cell proliferation (66). Similarly, Webb (67) *et al.* generated a modified form of A2M named ‘macroglobulin activated for cytokine binding’ or MAC. MAC was formed through consecutive reactions with the amino acid side-chain crosslinker cis-dichlorodiammineplatinum-II and MA. MAC had increased binding affinity with the pro-inflammatory cytokines TNF-α and IL-1β (67). Administration of MAC (intraperitoneal) prior to lipopolysaccharide (LPS)-challenge in mice (intravenous injection), increased the survival rate (67). In a model for peripheral nerve injury, MAC could also suppress inflammation. Hence, it has been suggested that MAC has an anti-inflammatory function through binding inflammatory cytokines (44). Interestingly, in a different study it was shown that oxidation of A2M or A2M** by hypochlorite (an oxidating agent secreted by neutrophils), increases the affinity of A2M and A2M** for TNF-α, IL-2, and IL-6, but decreases the affinity for β-NGF, PDGF-BB, TGF-β1, and TGF-β2. This implicates that oxidative modification of A2M during inflammation might alter the repertoire of A2M-binding cytokines and growth factors (46). Interestingly, mice deficient in A2M have significantly lower levels of plasma TNF-α and develop a short-term attenuated fever in response to LPS administration. Therefore, it has been suggested that the A2M chemokine-binding capacities might be important for the development of fever (68).

## The Endocytic and Signaling Receptors

### LRP-1/the A2M Receptor/CD91

**Figure 3:**
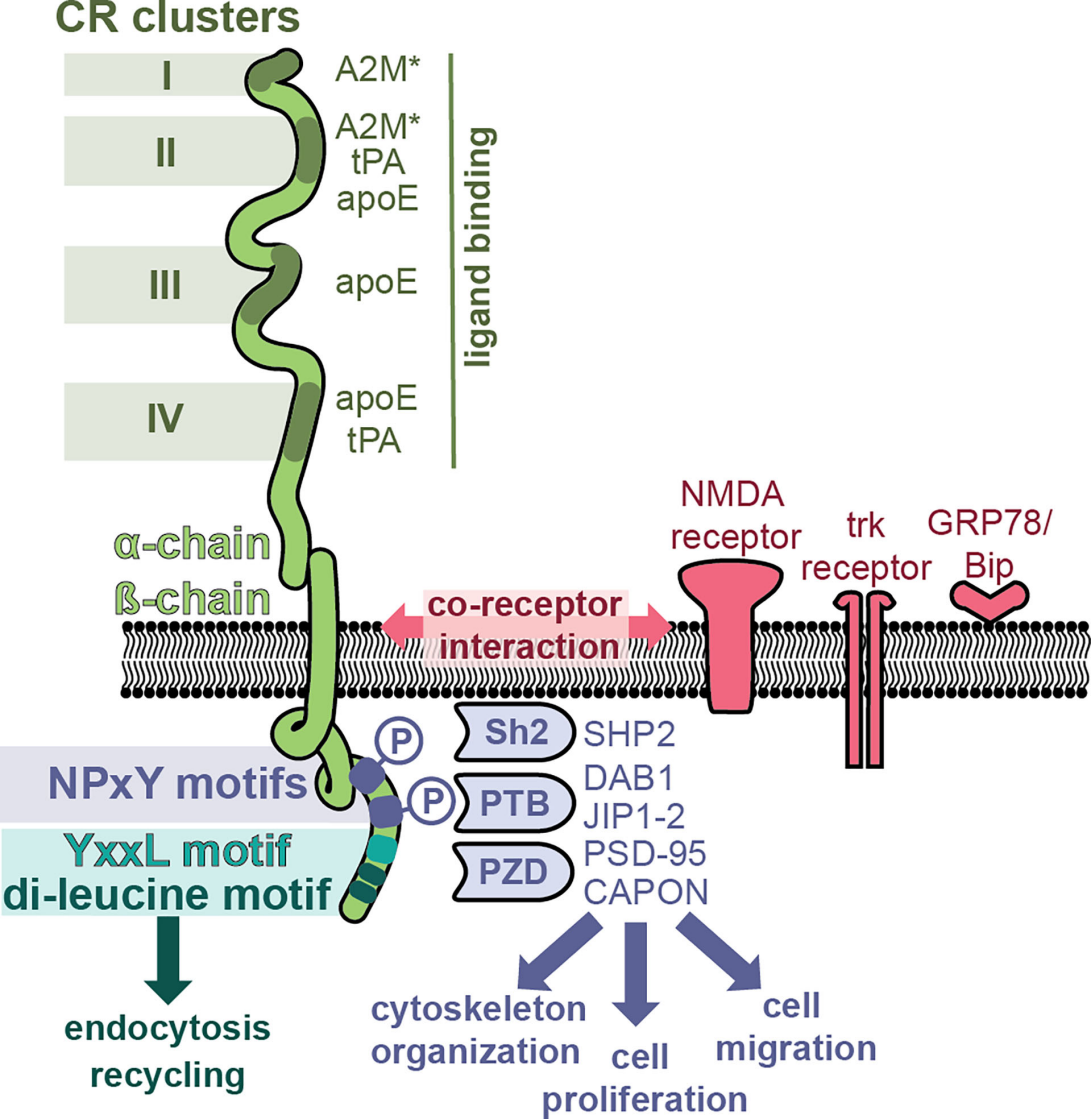
LRP-1, a multifunctional receptor for A2M*. The LRP-1 receptor (green) is composed out of a 515-kDa extracellular domain (α-chain) and an 85 kDa transmembrane domain. The α-chain has four regions with cysteine-rich complement-type repeat (CR) clusters (clusters I-IV) which interact with extracellular ligands such as A2M*, tissue plasminogen activator (tPA) and apolipoprotein E (apoE). The intracellular part of the β-chain initiates endocytosis and interacts with adaptor molecules to initiate signaling pathways. Whereas endocytosis is mediated through the YxxL motif and dileucine repeats, the initiation of cell signaling pathways relies on the presence and phosphorylation of NPxY motifs and recruitment of adaptor and scaffolding proteins containing phosphotyrosine-binding (PTB) domains [e.g. Disabled homolog 1 (DAB1), C-Jun-amino-terminal kinase-interacting protein 1 & 2 (JIP1-2)], Src homology 2 (Sh2) domains [e.g. Sh2-containing protein tyrosine phosphatase 2 (SHP2)] and PDZ (post synaptic density protein (PSD95), drosophila disc large tumor suppressor and zonula occludens-1 protein) domains [e.g. PSD-95, protein carboxy-terminal PDZ ligand of nNOS (CAPON)]. Hence, LRP-1 can activate different signaling pathways, thereby affecting processes such as cytoskeletal reorganization, cell proliferation and cell adhesion. In addition, several co-receptor relationships have been proposed, for example, with the N-methyl-D-aspartate (NMDA) receptor, the tyrosine receptor kinase (trk) receptor and glucose-regulated protein (GRP)-78.

LRP-1 (also called the A2M receptor or CD91) is a large, multifunctional receptor, composed out of a non-covalently bound 515-kDa extracellular domain (also called the ‘heavy domain’ or α-chain) and an 85 kDa transmembrane domain (also called the ‘light chain’ or β-chain) (**Figure 3**). These two domains are formed from a 600 kDa precursor protein after cleavage by furin (69). After its biosynthesis, LRP-1 interacts with a 39 kDa receptor-associated protein (RAP) which can also be found associated with LRP-1 at the cell surface (70). RAP efficiently blocks LRP-1 function and is therefore an endogenous regulator of LRP-1 activity (71). The extracellular LRP-1 α-chain holds four regions with cysteine-rich complement-type repeats (CR) (72), also referred to as clusters I-IV or ligand binding domains, and which are the main interaction interface for extracellular ligands. Clusters II and IV were identified as the most widely used interaction sites for LRP-1 ligands (73).

The best known function of LRP-1 is its role as a scavenging receptor for many proteins including protease, protease inhibitors, complement proteins and even toxins and viruses (74). After ligand binding, LRP-1 undergoes efficient endocytosis *via* clathrin-coated pits and subsequent recycling. This process is mainly mediated through the YxxL motif and dileucine repeats, present in the intracellular part of the LRP-1 β-chain (75) (**Figure 3**). Activated A2M* undergoes efficient cellular uptake through interaction with LRP-1. For example, in mouse fibroblasts (BaIb 3T3 cells), cellular uptake and presence of A2M in cytoplasmic vesicles occurs within 5 minutes, and after 15-30 minutes the content of these vesicles is found in the lysosomes (76). Upon proteolysis of the A2M bait region by active proteases or chemical activation, the A2M receptor binding domains (RBD, **Figure 1**) are exposed, which form the interface between LRP-1 and A2M*/A2M** (77). When evaluating variants of the receptor binding domain, two lysine residues (Lys^1393^ and Lys^1397^) in the A2M receptor binding domain were found to be crucial for receptor binding (78–80). Moestrup (81) *et al.* found that trypsin-A2M* can bind with low (kd = 2 nM) and with high (kd = 40 pM) affinity to LRP-1 and proposed that binding efficiency depends on the availability of the receptor (receptor density). For example, at low receptor density only one subunit of the A2M tetramer will interact with one LRP-1, whereas at high receptor density two or more LRP-1 receptors will bind per A2M tetramer (81). Electron microscopy visualization showed that up to three LRP-1 receptors could bind to chymotrypsin-A2M* (82). In addition, cooperative binding to several LRP-1 clusters has been reported, for example, A2M*-trypsin complexes cooperatively bind to cluster I and cluster II (83).

The LRP-1 β-chain also holds several signature sequences that connect LRP-1 with adaptor proteins involved in cell signaling and protein trafficking (84). Two NPxY domains are located in the C-terminal part of LRP-1 (**Figure 3**) and these provide binding sites for several adaptor and scaffolding proteins containing phosphotyrosine-binding (PTB) domains [e.g. disabled homolog 1 (DAB1), C-Jun-amino-terminal kinase-interacting protein 1 & 2 (JIP1-2)] (84) or proteins containing Src homology 2 (Sh2) domains [e.g. Sh2-containing protein tyrosine phosphatase 2 (SHP2)] (85). In addition, also several adaptor proteins containing PDZ (post synaptic density protein (PSD95), drosophila disc large tumor suppressor and zonula occludens-1 protein) domains were found to bind the LRP-1 intracellular tail [e.g. PSD-95, protein carboxy-terminal PDZ ligand of nNOS (CAPON)] (84). Hence, LRP-1 has the potential to activate different signaling pathways, driving processes such as cytoskeletal reorganization, cell proliferation, apoptosis and cell adhesion (84). Interestingly, it appears that phosphorylation of the distal NPxY site is mostly involved in the interaction with signaling proteins, whereas the proximal phosphorylation site is more involved in the process of receptor recycling, suggesting non-redundant properties (73, 84, 85).

Whereas the multifunctional potential of LRP-1 is clear, knowledge on how each of these pathways in triggered by different LRP-1 ligands and how LRP-1 selects among these pathways remains incomplete. One possible way is through the interaction with a co-receptor (86). For example, LRP1 functions as a single system with the N-methyl-D-aspartate (NMDA) receptor and tyrosine receptor kinase (trk) receptor, to activate cell signaling [extracellular signal−regulated protein kinase (ERK)-1/2] in response to tissue plasminogen activator (tPA) and A2M**. In contrast, myelin-associated glycoprotein affects LRP-1 differently and results in recruitment of p75 neurotrophin receptor (p75NTR) into a complex with LRP-1 and activated Ras homolog family member A (RhoA) (86).

LRP-1 has generally low tissue specificity and is expressed ubiquitously, including in hepatocytes, neurons, astrocytes, epithelial cells of the gastrointestinal tract, SMCs, fibroblasts, Leydig cells in testis, granulosa cells in ovary, and dendritic interstitial cells of the kidneys (87). In addition, monocytes and macrophages express more LRP-1 (88). One method of LRP-1 regulation is proteolytic shedding of its ectodomain from the cell surface. Membrane-associated proteases including membrane-type 1 matrix metalloproteinase (MT1-MMP) and a disintegrin and metalloprotease 17 (ADAM17) are capable of shedding LRP-1 in chondrocytes (89) and the prevalence of soluble LRP-1 (sLRP-1) was shown to correlate with pro-inflammatory conditions (90). Following LPS injection, increases in sLRP can be found in mouse plasma. In conditions of chronic inflammation, increased levels of sLRP-1 have also been found (90). Interestingly, stimulation of macrophages with purified sLRP results in a pro-inflammatory effect and activation of nuclear factor kappa-light-chain-enhancer of activated B cells (NF-κB), c-Jun N-terminal kinases (JNK) and p38 mitogen-activated protein kinases (p38 MAPK) (90). In contrast, other studies found anti-inflammatory functions for sLRP-1. For example, sLRP-1 could reduce the pro-inflammatory effects of TNF-α (91).

### GRP78/BiP/HSPA5

LRP-1 was identified as the first receptor for activated A2M, but the evidence for a second receptor was published in 1994 by Misra (12) *et al.* In this study, the LRP-1 inhibitor RAP could not block A2M**-mediated increases in intracellular calcium and inositol 1,4,6-triphosphate in macrophages, which led the authors to suggest the existence of a second receptor (12). The newly identified receptor functioned through a pertussis toxin-insensitive G-protein and contrasts with LRP-1 mediated signaling, which occurs through a pertussis toxin-sensitive G protein (12, 92). From the membrane fraction of mouse macrophages and 1-LN prostate cancer cells, GRP78 (78 kDa) was identified as the second A2M-receptor (13). This interaction was confirmed when purified GRP78 was found to bind A2M** with high affinity (Kd ~ 150 pM) (13). In addition, LRP-1 was co-purified during this study, suggesting a co-receptor relationship between GRP78 and LRP-1 (13) (**Figure 3**).

Surprisingly, GRP78 is mainly known as an intracellular protein and member of the heat shock protein 70 (HSP70) family involved in correct translocation and folding of newly synthesized polypeptides across the endoplasmic reticulum membrane (93). Glucose-regulated proteins are induced under conditions of cellular stress, such as glucose starvation or agents affecting calcium stores or glycosylation patterns (94). Consequently, GRPs are typically increased in conditions involving tissue starvation and stress such as ischemia, vascular dysfunction, inflammation, apoptosis and necrosis. Under healthy conditions they are thought to protect against cell death, whereas the anti-apoptotic functions might also support the survival of neoplastic cells and their resistance to treatments (94–96). Based on co-immunoprecipitation in macrophage membrane fractions, it was shown that DnaJ homolog subfamily C member 1 (Dnajc1, also called MTJ-1) interacts with GRP78 at the cell membrane and enables cell-surface localization of GRP78 (97). Dnajc1 belongs to the family of J domain proteins (JDPs) which bind and activate Hsp70 proteins through their J domain (98). The interaction of GRP78 with Dnajc1 might thus explain the association of GRP78 with cell membranes.

Functionally, it was shown that A2M** signaling through GRP78 triggers pro-proliferative and anti-apoptotic behavior in macrophages and cancer cells (99). In prostate cancer cells, binding of A2M** to GRP78 causes an increase in prostate-specific antigen (PSA). PSA is secreted as an active serine protease which again binds and activates more A2M, resulting in a positive feedback loop where PSA-A2M* complexes bind GRP78, activate mitogen-activated protein kinase (MEK)-1/2, ERK1/2, S6 kinases S6, and Akt resulting in the promotion of DNA and protein synthesis and increased cell proliferation (100). A detailed overview of A2M*/A2M** signaling through GRP78 on macrophages is provided in the next chapter.

## Effects of A2M on Leukocytes

In this section we will discuss the effects of A2M on leukocytes, specifically neutrophils, monocytes, macrophages, and lymphocytes. Generally, the roles of A2M in leukocyte biology relate to all previously mentioned aspects of A2M function including inhibition of protease activity, binding of immunologically important molecules (e.g. cytokines/growth factors) and binding of cell-surface receptors for clearance or to trigger diverse pathways relevant for cell function.

### Neutrophils

**Figure 4:**
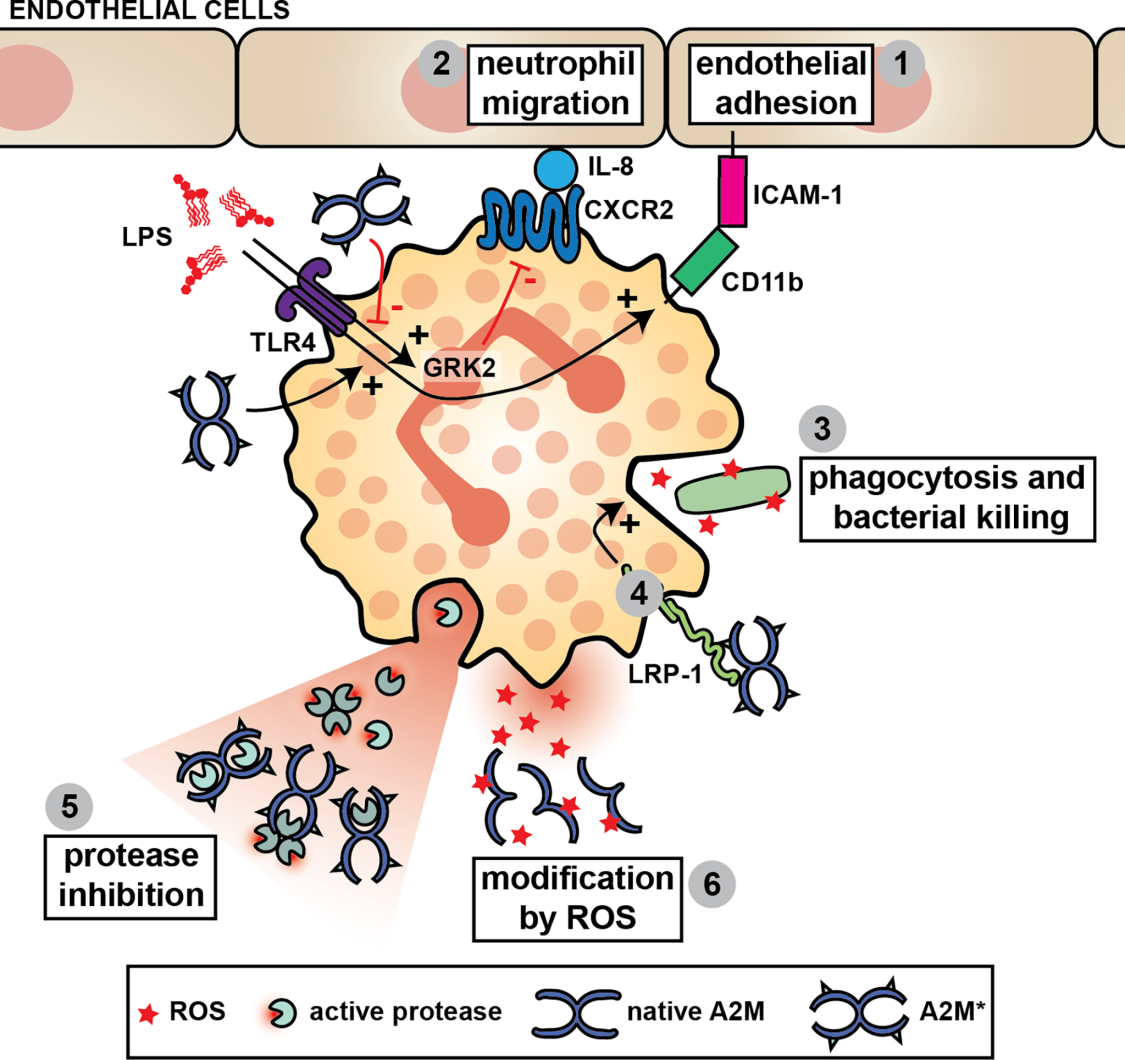
A2M and neutrophil function. A2M* aids neutrophils through stimulation of their capacity to bind to endothelial cells (**1**), to migrate (**2**) and to phagocytose and kill pathogens (**3**). For example, the chemotactic potential of neutrophils is increased in the presence of A2M* by reducing LPS-induced G-protein-coupled receptor kinase 2 (GRK2) expression, which subsequently results in increased C-X-C motif chemokine receptor 2 (CXCR2) expression and increased neutrophil migration towards IL-8 (**2**). A2M* also induces CD11b in neutrophils challenged with LPS, thereby increasing the interaction with ICAM-1 and aiding neutrophil adhesion to the vascular endothelium (**1**). A2M* augments phagocytosis and bacterial killing by neutrophils, possibly through interaction with LRP-1 (**4**). Finally, A2M can inhibit all major proteases secreted by neutrophils and reduce their proteolytic activity against large substrates (**5**). However, in the presence of reactive oxygen species (ROS), such as hypochlorite, A2M dissociates thereby switching its activity from protease inhibition to an extracellular chaperone and cytokine carrier (**6**).

A2M inhibits the activity of the main proteases released from stimulated human neutrophils (101), specifically NE (101–104), proteinase 3 (P3) (105), cathepsin G (catG) (101) and matrix metalloproteinase-9 (MMP-9) (106, 107). However, inhibition of total neutrophil proteolysis - in *in vitro* assays with isolated neutrophils or their full degranulates - is often incomplete (102, 103). For example, only 73.5% of fibronectin proteolysis by neutrophils can be inhibited by A2M (102). Several explanations for this phenomenon have been proposed. First, proteases trapped by A2M remain functionally active against substrates that can access the interior of the A2M molecular cage (104). For example, in bronchoalveolar lavage fluid from patients with adult respiratory distress syndrome, NE activity against a low molecular weight substrate could be measured despite a 30-fold excess of the anti-proteases alpha-1-antitrypsin and A2M. Whereas most NE was complexed to these inhibitors, NE activity against small substrates could be associated with NE-A2M* complexes (104). Second, as part of the antimicrobial host defense, stimulated neutrophils produce and release a range of reactive oxygen species (ROS) which act by oxidizing and modifying biological components. Therefore, it was suggested that neutrophils inactivate A2M by the release of reactive species such as hypochlorite. Through oxidative modification, ROS affect A2M structural integrity and cause dissociation of A2M tetramers into dimers which do not possess anti-proteolytic activity (108–111) (**Figure 4**). Finally, certain proteases partially escape regulation by A2M. For example, MMP-9, a metalloproteinase secreted from neutrophil specific granules, is produced as both a monomeric and homotrimeric form. In contrast to monomeric MMP-9, trimeric MMP-9 is able to bind A2M and remain active against large substrates, likely due to its size difference (107) (**Figure 4**).

Whereas ROS such as neutrophil-derived hypochlorite might revoke the ability for A2M to capture and inhibit proteolytic enzymes, other important implications recently have been discovered. First, hypochlorite alters the cytokine/growth factor binding profile of A2M and A2M** (46). This modification increases the affinity of A2M and A2M** for TNF-α, IL-2, and IL-6, but decreases binding to β-NGF, PDGF-BB, TGF-β1, and TGF-β2. Hence, it was proposed that oxidation would decrease the progression of acute inflammation by sequestering TNF-α, IL-2, and IL-6, while up-regulating the development of tissue repair processes by releasing factors important for healing (46). Second, it was shown that A2M exposed to hypochlorite dissociates into stable dimers with potent chaperone activity for damaged proteins. For example, hypochlorite-induced dimers efficiently inhibit heat-induced aggregation of creatine phosphokinase and citrate synthase. Furthermore, these dimers also bind amyloid β-peptide (Aβ1–42) and facilitate its removal through lipoprotein receptors, thereby reducing neurotoxicity (112, 113). Once converted to A2M* by reaction with proteases, these complexes undergo efficient receptor-mediated uptake (e.g. by monocyte/macrophage RAW 264.7 cells), thereby avoiding the build-up of toxic protein aggregates (112, 114).

In human neutrophils, LRP-1 is found at the cell surface as well as intracellularly. Upon stimulation with LPS, LRP-1 is mobilized from intracellular stores to the cell surface, which suggests a role for LRP-1 in anti-microbial defense (115). Interestingly, A2M is also a major component of neutrophil-derived microparticles. In a proteomics study of neutrophil-derived microparticles, microparticles of human umbilical vein endothelial cell (HUVEC)-adhering neutrophils were found to be enriched in A2M (116). In the presence of active A2M, LPS-challenged neutrophils increasingly expose the adhesion and migration marker CD11b. This results in increased interaction with ICAM-1, likely increasing neutrophil adhesion to vascular endothelium (**Figure 4**). Likewise, A2M is thought to improve the capacity of endothelial cells to interact with neutrophils. Pretreatment of TNF-α-stimulated HUVECs with A2M-enriched microparticles or microcapsules also results in an increase in neutrophil adhesion onto the endothelial cell monolayer (115, 117). No difference in the expression of endothelial adhesion molecules was found, but it was suggested that A2M is delivered onto the endothelial cells plasma membranes where it engages LRP-1 on the neutrophils promoting firm leukocyte adhesion (115).

*In vitro*, A2M enhances the migration of polymorphonuclear cells towards neutrophil-derived eosinophil chemotactic factor (118). Activated A2M quickly (1h stimulation) reduces LPS-induced G-protein-coupled receptor kinase 2 (GRK2) and increases CXCR2 leading to increased migration of LPS-stimulated neutrophils towards IL-8 (115). In agreement with these findings, administration of A2M microvesicles prior to a cecal ligation puncture (CLP) procedure in mice (as a model for polymicrobial sepsis) resulted in an increase of early (6h) neutrophil recruitment (115). Hence, this confirms that A2M is chemokinetic in neutrophils (119).

Finally, A2M has also been implicated in neutrophil phagocytosis and bacterial killing. A2M binds to the surface of *Streptococcus pyogenes* (groups A, C and G) (120, 121). So far, the identified protein interaction partners are either the N-terminal region protein G (groups C and G) (122) or the protein G-related A2M-binding protein (GRAB) (group A) (123). Whereas earlier studies report that binding of A2M to *S. pyogenes* enhances its phagocytosis by human neutrophils (124), more recent studies propose that *S. pyogenes*-bound A2M protects this bacteria against host proteases and is thus a virulence factor in *S. pyogenes* infections. In a separate study, neutrophils pre-treated with activated A2M or A2M-enriched microparticles, had an increased capacity to phagocytose *E. coli* and produced increased amounts of ROS and bactericidal cathelicidins, a process which was LRP-1-dependent (115).

### Monocytes and Macrophages

**Figure 5:**
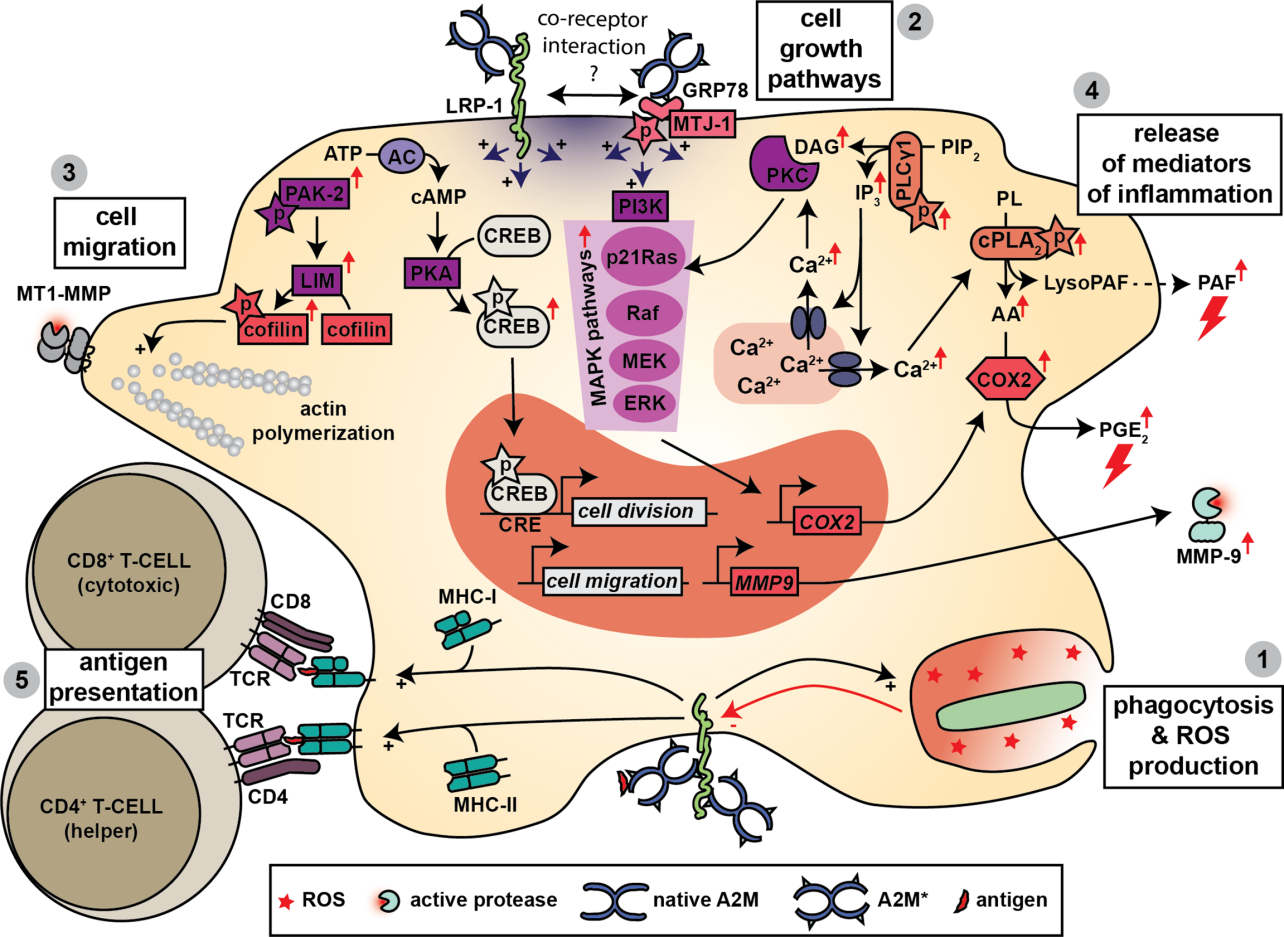
A2M and macrophage function. Macrophages produce A2M and express both A2M receptors. Hence, several functions for A2M have been proposed in macrophage biology. A2M promotes bacterial phagocytosis, killing and the production of ROS (**1**) and this effect might be mediated through interaction with LRP-1. Triggering of LRP-1 and/or GRP78-associated signaling pathways leads to several cellular effects. A first effect is increased cell growth, which is mediated through activation of MAPK pathways, phosphorylation of cAMP response element-binding (CREB) protein and activation of protein kinase C (PKC) (**2**). A second effect is increased cell migration, which might occur through increased phosphorylation of cofilin, activation of actin polymerization and the formation of enlarged cellular protrusions containing MT1-MMP (**3**). Binding to LRP-1 and/or GRP78 also results in a rapid increase in intracellular calcium which is thought to contribute to the release of mediators of inflammation including platelet activating factor (PAF), prostaglandin E2 (PGE2) and matrix metalloproteinase-9 (MMP-9) (**4**). Finally, A2M also promotes antigen presentation by macrophages where both MHC-I and MHC-II presentation have been reported (**5**).

Macrophages are specialized in sensing and attacking invading pathogens, in modulating the immune response by secretion of inflammatory mediators and finally in contributing to healing of damaged tissues (125). Given that macrophages secrete A2M (126, 127) and also express both A2M receptors (128–130) it is not surprising that protease-A2M* complexes are efficiently taken up by macrophages (131) and that several roles for A2M* in macrophage function have been proposed (**Figure 5**).

Comparable to neutrophils, A2M increases the phagocytic and anti-microbial capacity of macrophages. For example, *Trypanosoma cruzi*, the parasite causing Chagas’ disease, undergoes more efficient uptake by mouse macrophages when pre-treated with A2M (132). Human monocyte-derived macrophages pre-treated with activated A2M or A2M-enriched microparticles or microcapsules also increase bacterial phagocytosis (*E. coli*), phagocytosis of zymosan and ROS production (115, 117). This effect can be blocked by anti-LRP-1 antibodies and is thus thought to be mediated through LRP-1 (115). Interestingly, several lines of research support the idea that the activation of anti-bacterial or antiviral pathways in macrophages leads to the suppression of A2M function and its associated pathways. For example, endotoxin stimulation suppresses A2M secretion, and stimulation with LPS or interferon (IFN)-γ results in a reduced mRNA expression of LRP-1 (128, 133). In the macrophage-derived cell line J774, effects triggered by A2M** and occurring though LRP-1, could also be abrogated when cells were first challenged with LPS (134). Even microglia, the resident macrophages of the central nervous system, express LRP-1 and internalize A2M**, a process which is decreased upon stimulation with LPS or IFN-γ (135).

In a series of studies on mouse macrophages, Misra *et al.* performed a detailed analysis of the cell signaling cascades activated by A2M*/A2M** and mediated through GRP78. When stimulated with A2M**, mouse macrophages undergo a rapid increase in intracellular Ca^2+^ and in signaling molecules such as inositol trisphosphate (IP3), diacyl glycerol (DAG), arachidonic acid (AA), lysophosphatidylcholine (lysoPC) and cyclic adenosine monophosphate (cAMP) (14, 136). In addition, a gradual increase in cytosolic pH occurs (130). Several pathways were identified including phosphorylation of phospholipase Cγ1 (PLCγ1) and an increase in phosphorylation, activity and membrane/nuclear translocation of cytosolic phospholipase A2 (cPLA2) (130, 137, 138). Activation of several mitogen−activated protein kinase (MAPK) cascades was also suggested based on the finding of increased phosphorylation of MEK 1/2, ERK 1/2, p38 MAPK, and JNK (137). Phosphorylation of the transcription factor cAMP response element-binding (CREB) protein was also reported (139). Activation of these pathways may have several important consequences for macrophage biology. For example, increased protein and DNA synthesis was reported, which improved cell division (139, 140). Synthesis and metabolism of platelet activating factor (PAF) was altered, resulting in increased release of this potent inducer of acute inflammation (141). Typsin-A2M* and A2M** could also induce the secretion of eicosanoids [e.g. prostaglandin E2 (PGE2)] from cultured mouse macrophages (142, 143). This occurs through upregulation of total cellular and nuclear cyclooxygenase-2 [COX2, also known as prostaglandin-endoperoxide synthase-2 (PTGS2)], and requires participation of both the p21-ras dependent MAPK and PI 3-kinase signaling pathways (144). Finally, given the fact that these pathways were resistant to inhibition by the LRP-1 antagonist RAP, it was suggested that these are triggered mainly through the GRP78 receptor (12, 92, 130). In addition, silencing of LRP-1 did not have an effect on A2M**-induced IP3 synthesis (13). Nevertheless, based on the co-immunoprecipitation of GRP78 with LRP-1 a co-receptor relationship was also suggested (13). In a different study increased cell proliferation by A2M** (in the J774 macrophage-derived cell line) also relied on MAP kinase phosphorylation (Mek1-ERK1/2 pathway), but was dependent on LRP-1 (134). Furthermore, in a follow-up study it was shown that A2M** also induces the expression and secretion of MMP-9 and involved PKC and extracellular calcium influxes (145).

A2M can also influence macrophage morphology and their capacity to migrate. For example, A2M*/A2M** are able to counter IFN-γ-induced morphological changes in mouse peritoneal macrophages (146). Furthermore, in a macrophage-derived cell line (Raw264.7 cells), A2M** increased cell migration in an *in vitro* wound-migration assays, and induced the formation of enlarged cellular protrusions containing MT1-MMP. This process was dependent on LRP-1 and was mediated through activation of PKC (147). In contrast, in a study by Misra *et al.*, mouse macrophages stimulated with A2M** had increased tyrosine phosphorylation of GRP78, autophosphorylation of p21-activated protein kinase-2 (PAK-2), phosphorylation of LIM kinase and cofilin, causing cytoskeletal rearrangements (148).

Finally, a role for A2M in promoting antigen presentation by macrophages was suggested. Antigens conjugated to A2M are efficiently taken up by mouse macrophages which results in efficient activation of murine T-cells, which suggests that A2M could aid in antigen presentation (149). For example, hen egg lysozyme (HEL), complexed to elastase-A2M* (ELA-A2M*-HEL), is taken up by mouse macrophages more efficiently than free HEL, a process which relies on receptor-mediated endocytosis. Subsequently, the threshold for antigen presentation to (and activation of) HEL-specific T-hydridoma cells was 2.2 to 2.7 log units lower than free HEL (15). In rabbits, subcutaneous injection of ELA-A2M*-HEL complexes also resulted in 10 to 500-fold higher IgG titers compared to uncomplexed controls (150). Furthermore, this adjuvant-like effect could also be established with microbial antigens. Proteins isolated from Kirsten murine sarcoma virus and subsequently conjugated to A2M (by activation with trypsin), were more efficiently taken up by mouse thioglycolate-induced peritoneal exudate cells compared to unconjugated viral proteins. Subsequent co-culturing with spleen cells resulted in higher amounts of antibodies against viral proteins (151). Similarly, complexes between cruzipain, a cysteine protease from *Trypanosoma cruzi*, and A2M* were more efficiently taken up by human monocytes and resulted in enhanced (MHC-II-dependent) presentation of cruzipain peptides to CD4+ T cells from patients witch Chagas’ disease (152).

A new insight in A2M-mediated antigen presentation was achieved with the identification of LRP-1 as the receptor for the heat shock protein gp96/CD91 (153). Gp96 is known for its immunogenicity through antigen binding, followed by uptake and MHC-I dependent presentation by antigen presenting cells. Given the striking similarities with A2M, the same research group subsequently confirmed that both gp96 and A2M act as T-cell adjuvants that can facilitate the transition of exogenous antigens into the endogenous pathway for antigen presentation (154). This effect was corroborated in a study where an A2M**-delivered antigen enhanced the expansion of a CD8+ T cell population, resulting in a 25-fold greater secretion of IFN-γ and IL-2, and induced cell-mediated cytotoxicity (16). Hence, both MHC-I and MHC-II-dependent processes can be promoted by antigen conjugation to A2M.

### Lymphocytes

As discussed previously, binding to activated A2M increases the ability of antigens to be presented to T-cells by macrophages and this can happen both in MHC-I and MHC-II context (16, 149–152, 154). Hence, A2M can have an indirect effect on lymphocyte proliferation. In addition, A2M can bind several cytokines, including IL-2 which is a produced by activated T-cells. Due to its small size, the cytokine IL-2 can still be degraded by A2M*-captured trypsin, resulting in the loss of its capacity to stimulate the proliferation of mouse cytotoxic T lymphocytes (CTLL-20 cells) and primary human lymphocytes (155, 156). A2M can capture proteases ranging from approximately 20 to 100 kDa and allows substrates of up to approximately 17 kDa to enter its molecular cage (depending on their overall structure) (4). Hence, the proteolytic activity of A2M-bound proteases remains highly relevant in the context of immune-modulation by small soluble cytokines. Given the important role for cytokines such IL-2 in lymphocyte function, one might wonder what the net contribution of A2M is taking the potential proteolysis and degradation of cytokines into account. Furthermore, A2M might switch proteolysis to smaller substrates while inhibition of large or cell surface-bound substrates remains intact. To that regard, Petersen *et al.*, reported that native A2M blocks T-cell-mediated cytotoxicity and that this effect relies on the anti-proteolytic activities of A2M (157). Direct effects of A2M on lymphocyte biology have, however, not yet been described. Perhaps this is due to the fact that the expression of the LRP-1 receptor in T-cells and B-cells is limited, albeit, recent RNA-Seq studies profiling human PMBC immune subsets demonstrated an expression level of LRP-1 similar (or even higher than) neutrophils in certain T-cell and B-cell subsets (87, 158).

## A2M in Inflammation and Infection

In this part we discuss the established relevance of A2M in a selection of immune-mediated pathologies and its contribution to infections. Most disease-related studies focus on the ability of A2M to inactivate active proteases and its ability to bind cytokines, whereas other contributions of A2M remain unexplored.

### A2M in Sepsis Syndromes

In patients with sepsis or animal models for sepsis, plasma native A2M is decreased whereas activated A2M is present at higher levels. This change is likely due to the formation of complexes with proteases released by activated neutrophils or proteases that are part of the fibrinolytic or coagulation cascade. Hence, complexes such as plasmin-A2M*, thrombin-A2M*, cathepsin-G-A2M*, and elastase-A2M* can be found (159, 160). In guinea-pigs, depletion of A2M results in high lethality upon administration of pseudomonal elastase or *Pseudomonas aeruginosa* culture supernatants, an effect that appears to rely on activation of coagulation factor XII (also known as Hageman factor), which is a serine endopeptidase part of the coagulation cascade. Furthermore, restoration of A2M levels could abrogate this effect (161). In a different study, A2M-deficient mice were also more sensitive to endotoxin, but induction of a lethal Gram-negative infection with *Klebsiella pneumoniae* rendered A2M-deficient mice more resistant. A2M-deficient mice more efficiently cleared *K. pneumoniae* from major organs in comparison with control mice (162). This finding is highly surprising given that fact that A2M promotes phagocytosis and killing of bacteria by neutrophils and macrophages.

Microparticles enriched with A2M (as produced by adherent neutrophils) are found in plasma samples from patients suffering from severe sepsis and vary according to the source of infection (116, 163). A2M-containing microvesicles are associated with survival in community acquired pneumonia-associated sepsis, but not with sepsis caused by fecal peritonitis (163). Furthermore, the amount of A2M-containing microparticles in plasma from sepsis survivors is higher than in plasma from non-survivors and healthy volunteers (116). In the CLP mouse model for bacterial sepsis, administration of soluble A2M or A2M-enriched human microvesicles (A2M-E-MV) improves survival rates. A2M-E-MV also protects against hypothermia and reduces bacterial loads by improving bacterial phagocytosis. Surprisingly, both A2M and A2M-E-MV reduce total proinflammatory lipid mediator levels (e.g. PGE2), increase IFN-γ levels, and A2M-E-MV alone increase pro-resolving lipid mediator levels. Furthermore, mice receiving A2M-E-MV had lower levels of peritoneal leukocytes and lung myeloperoxidase levels (115). Finally, a conjugate between the LPS-binding antibiotic Polymyxin B and A2M was also able to decrease the lethality of both LPS-induced acute inflammation and polymicrobial sepsis induced by cecal ligation and puncture (CLP) in mice, when administered before or shortly after start of the model (164). Overall, it appears that supplementation with A2M or A2M-microparticles, has beneficial outcomes in sepsis and might have therapeutic potential in these syndromes.

### Lung Inflammation

Lung inflammation is characterized by abundant secretion of extracellular proteases which contribute to lung damage (165). A logical role for A2M is therefore the inhibition of excessive proteolysis. Indeed, during acute respiratory distress syndrome (ARDS), A2M binds active NE. However, whereas A2M-captured NE is no longer able to cleave insoluble elastin, it remains active against small substrates and this activity can no longer be inhibited by alpha-1-antitrypsin (104). NE captured by A2M might therefore no longer contribute to connective tissue injury, but is still able to modify smaller substrates such as cytokines. This again supports the hypothesis that A2M switches proteolysis towards the catalysis of small substrates. Another particularly interesting discovery is the finding of complexes between IL-8 and activated A2M in lung fluids from patients with ARDS. Whereas these complexes might not be detectible by standard IL-8 immunoassays, IL-8 complexed to A2M retains its ability to attract and activate neutrophils and even protects IL-8 against proteolysis by NE (48, 166). As a result, the complexes might account for an underestimation of biologically active IL-8.

### Rheumatic Diseases

In the inflamed joint, A2M is associated with synovial fluid monocytes, cells of the synovial lining and perivascular cells. Furthermore, the abundancy of A2M correlates with the degree of inflammation (167–169). Levels of inactive A2M correlate with neutrophil numbers and are thought to be generated by intraarticular reaction with proteases or by oxidating agents. Whereas A2M is found complexed to elastase-like and chymotrypsin-like proteases (presumably NE and cathepsin G), the majority of inactive A2M is thought to be generated through reaction with ROS (170). A first consequence of A2M oxidation is the decreased capacity of oxidized A2M to inhibit proteases. Synovial fluids from patients with rheumatoid arthritis (RA) contain a 2-fold higher amount of A2M compared to that from osteoarthritis (OA) patients, but the amount of oxidized A2M in RA is significantly higher and the ability of A2M to inhibit proteolysis is lower than that with OA (111). A2M oxidation also influences the capacity of A2M to bind cytokines and growth factors. It has also been proposed that A2M oxidation down-regulates inflammation by shifting the A2M cytokine/growth factor binding profile towards TNF-α, IL-2, and IL-6, while up-regulating the development of tissue repair by reduced binding to bFGF, β-NGF, PDGF, and TGF-β. To that regards, it was shown that A2M from synovial fluid of RA patients, had a decreased capacity to bind TGF-β compared to A2M from synovial fluid of OA patients (46). Finally, hypochlorite-induced oxidation of A2M also induces the chaperone function of A2M. It remains to be determined what the contribution of this effect would be in arthritis.

Another finding is that NE-A2M* complexes can still degrade proteoglycan in sections of human articular cartilage (171). Furthermore, injection of collagenase-A2M* or trypsin-A2M* complexes into the joint cavity of healthy rabbits, also causes experimental synovitis which is more severe than trypsin or collagenase alone (167). Nevertheless, administration of A2M appears to have merit. In rats undergoing anterior cruciate ligament transection, intra-articular injection of A2M or A2M variants (with altered bait regions) attenuates OA damage, presumably by inhibiting cartilage degrading enzymes (172). In a collagen II‐induced arthritis model in mice, injection of A2M into the ankles, significantly reduced ankle thickness and improved disease scores (173). Finally, miR-146b, a microRNA associated with OA, was shown to negatively regulate A2M again supporting an overall beneficial role for A2M in arthritis (174).

### Infections

Given the unique mechanism and the broad substrate/inhibition repertoire of A2M, it is no surprise that it also binds proteases from microbial origin such as *Vibrio vulnificus* derived metalloprotease (175) and *Trypanosoma cruzi* cruzipain (176, 177). Cruzipain is the main cysteine protease present in all life-cycle stages of *T. cruzi* and in the presence of A2M, *T. cruzi* is more efficiently taken up by mouse macrophages (132). Furthermore, A2M also reduces *T. cruzi*-induced apoptosis of host cells and A2M** reduces DNA fragmentation of infected mouse macrophages (178). Finally, mice surviving *T. cruzi*-infection have higher levels of A2M compared to non-surviving mice (179). Whereas mice deficient in A2Ms have a lower blood parasitemia, analysis of mouse hearts revealed more amastigote nests and inflammatory infiltrates (180). Altogether, this suggests a protective role for A2M in *T. cruzi* infection.

A2M can also directly bind to pathogens and thereby influence the course of infections. A2M binds to the cell wall of *Streptococci* through binding of protein GRAB, a cell surface protein discovered on *Streptococcus pyogenes* and several clinical isolates. Surprisingly, A2M bound to *S. pyogenes* GRAB remains capable of inhibiting microbial and host proteases. Given that *S. pyogenes* mutants lacking GRAB have reduced virulence, it is hypothesized that *S. pyogenes* uses A2M as a protecting factor against host proteases (123, 181). This hypothesis was confirmed by the discovery that SpeB, a cysteine protease secreted by *S. pyogenes*, binds GRAB-bound A2M and protects these bacteria against the host antibacterial peptide LL-37 (182).

A role for A2M in the innate immune defense against viruses has also been proposed. In a proteomics study to identify the components of saliva responsible for inhibition of the H1N1 swine origin influenza A virus (virus-induced erythrocyte hemagglutination assay), A2M was identified as an essential inhibitor. The proposed mechanism involves the inhibition of host proteases responsible for influenza virus hemagglutinin activation and competition with the virus for binding 2,6-sialylated glycoprotein receptors on the host (183). Recently, it was also hypothesized that A2M might confer some protection from COVID-19 through its ability to protect the vascular endothelium and potential antithrombin activity. In addition, children have considerably higher plasma levels of A2M, which might correspond to the fact that children remain relatively resistant to severe COVID-19 (184, 185). However, the analysis of A2M protein levels in plasma samples from patients with COVID-19 revealed no significant differences or correlations with other disease parameters (186).

## Discussion

A2M is a unique macromolecule that interacts with a broad range of endopeptidases. Based on this feature and its ability to inhibit these proteases, its potential biological relevance is enormous. During inflammation, A2M protects against structural damage by inhibition of proteases released by activated leukocytes (e.g. neutrophils) (172). At the same time, A2M also inhibits proteases secreted by invading microorganisms (161). While A2M is mainly known as a general protease inhibitor, the discovery of many exceptions to this principle has indicated that A2M function is far more sophisticated than just protease inhibition. The mechanism through which A2M imposes protease inhibition provides the first clue for other important functions of A2M. A2M inhibits active proteases by forming a molecular cage around the protease and shielding the protease from its substrates (**Figure 1**) (2). However, this also implies that small proteins with the ability to access the A2M cage can still be subject to proteolysis (3). At the same time, the A2M cage also protects proteases from further inhibition by other protease inhibitors. For example, NE captured by A2M is no longer able to cleave insoluble elastin but remains active against small substrates. In addition, this activity can no longer be inhibited by alpha-1-antitrypsin (104). Whereas this finding is particularly important for conditions involving lung inflammation, similar effects can be anticipated in other conditions where major effects relate to the activity of small proteins. As an example, inflammatory chemokines are very susceptible to proteolysis and this modification can inactivate or even increase chemokine activity (187). Hence, it remains to be determined what the contribution of A2M could be in processes relying on chemokine activity. Along the same line, A2M (in particular A2M*) can bind to several cytokines and growth factors. Whereas this interaction seems to have no influence on IFN-γ, IL-1β, IL-8 and IL-6 activities, binding to FGF2, PDGF, TGF-β1 and TGF-β2 results in functional inhibition (see **Table 1**). Furthermore, for IL-6 and IL-8, A2M was shown to protect against proteolytic inactivation (47, 48). For many other cytokines and growth factors the functional relevance of this interaction remains to be determined. In addition, oxidative modification of A2M also alters the cytokine/growth factor binding capacity of A2M, presumably lowering pro-inflammatory mediators and aiding tissue healing (46).

In *in vitro* assays, A2M contributes to phagocytosis and killing of bacteria by neutrophils and macrophages. In both cell types, A2M increases the production of ROS or facilitates phagocytosis either indirectly through binding to the bacterium or directly through interaction with LRP-1 (115). Surprisingly, in A2M-deficient mice, *K. pneumoniae* was cleared more efficiently from major organs in comparison with control mice (162). Hence, the ‘net’ contribution of A2M in active bacterial infections *in vivo* remains to be determined. Furthermore, the exact contribution of A2M to pathogen clearance might also be pathogen-dependent. For example, in case of *S. pyogenes*, A2M functions as a virulence factor by binding to the bacterial surface and by capturing the bacterial protease SpeB and using it as protection against antibacterial peptides produced by the host (182).

Another shared contribution of A2M to neutrophil and macrophage function relates to increasing their motility (**Figure 4**). In macrophages, A2M* mainly appears to induce chemokinesis by activation of signaling pathways associated with cytoskeletal rearrangements (147, 148). In neutrophils, this effect mainly relates to chemotaxis and endothelial cell adhesion by inducing or stabilizing the presence of CXCR2 and CD11b (**Figure 3**) (115). These functions appear to be mediated through LRP-1, GRP78 or a combination of both receptors acting in a co-receptor relationship.

One discrepancy between *in vitro* and *in vivo* functions of A2M relates to the secretion of mediators of inflammation. *In vitro* stimulation of macrophages with A2M*/A2M** resulted in increased production of predominantly pro-inflammatory molecules such as PAF, PGE2 and MMP-9 (141–143, 145). However, in a mouse model for sepsis, administration of A2M reduced total proinflammatory lipid mediator levels (e.g. PGE2) (115). Hence, further investigation seems necessary. Administration of A2M, in particular A2M-enriched microparticles, appears to be beneficial in models for sepsis and increased levels of A2M are associate with better outcomes for sepsis patients. Hence a therapeutic use for A2M in sepsis syndromes or other types of systemic inflammations was suggested (188). Another potential application for A2M relates to its ability to enhance antigen presentation by macrophages. Antigens bound to A2M* are more efficiently presented to T-cells by macrophages. Meanwhile, a technique for the rapid and efficient incorporation of non-proteolytic antigens into A2M was developed (tradename, SynerVaxTM) and this technique was proposed as a novel adjuvant technology for vaccine development or antibody production (189).

The recent discovery of hypochlorite-treated A2M as an extracellular chaperone is particularly interesting in the context of neutrophilic inflammations (113). Hypochlorite secreted by activated neutrophils results in oxidation of A2M and its dissociation into dimers. Whereas these dimers no longer have the capacity to capture active proteases, they are able to form stable complexes with other ‘stressed’ proteins generated by oxidative modification. Subsequently, A2M aids their removal through receptor-mediated endocytosis (112). These mechanism might contribute to our understanding of how disordered proteins or cell debris generated by aggressive inflammatory environments are cleared and how build-up of pathological protein aggregates are avoided (19). Finally, being a highly conserved protein sharing structural similarities with complement factors C3 and C4 (23), it is interesting to see how some of the functions of A2M might relate to functions of the complement system such as opsonization and complement-mediated phagocytosis (190).

In conclusion, the potential contribution of A2M to inflammation, immunity and infection is clear. However, more studies are needed to understand which are the major mechanisms through which A2M contributes to pathology. To that regard, mechanistic studies using up-to-date technologies or applying *in vivo* models with cell specific deletion of A2M might provide crucial new insights and lead to new applications for A2M or new A2M-derivatives.

## Author Contributions

JV wrote the manuscript and YI provided crucial feedback and modifications. All authors contributed to the manuscript and approved the submitted version.

## Funding

This research was supported by the Research Foundation Flanders/FWO-Vlaanderen (G0A3820N) and the Belgian Charcot Foundation (JV). JV is supported by a senior postdoctoral fellowship of the Research Foundation of Flanders (FWO Vlaanderen, mandate 12Z0920N).

## Conflict of Interest

The authors declare that the research was conducted in the absence of any commercial or financial relationships that could be construed as a potential conflict of interest.

## Publisher’s Note

All claims expressed in this article are solely those of the authors and do not necessarily represent those of their affiliated organizations, or those of the publisher, the editors and the reviewers. Any product that may be evaluated in this article, or claim that may be made by its manufacturer, is not guaranteed or endorsed by the publisher.

## Glossary

**Table:**

A2M	alpha-2-macroglobulin
A2ML1	A2M-like-1
AA	arachidonic acid
ADAM17	a disintegrin and metalloprotease 17
ANA	anaphylatoxin domain
ApoE	apolipoprotein E
BiP	binding immunoglobulin protein
BRD	bait-region domain
C345C	C-terminal extension of C3-5
Uegf	Bmp1
cAMP	cyclic adenosine monophosphate
CatG	cathepsin G
CLP	cecal ligation puncture
COX2	cyclooxygenase-2
cPLA2	cytosolic phospholipase A2
CR	complement-type repeats
CUB	complement C1r/C1s
CXCR2	C-X-C motif chemokine receptor 2
FGF	fibroblast growth factor
DAB1	Disabled homolog 1
DAG	diacyl glycerol
GPI	glycosyl-phosphatidyl-inositol linker
GRAB	G-related A2M-binding protein
GRK2	G-protein-coupled receptor kinase 2
GRP	cell surface glucose-regulated protein
HEL	hen egg lysozyme
HSP70	heat shock protein 70
IL	interleukin
IP3	inositol trisphosphate
JDP	J domain protein
JIP	C-Jun-amino-terminal kinase-interacting protein
JNK	c-Jun N-terminal kinases
LysoPC	lysophosphatidylcholine
LRP-1	low density lipoprotein receptor–related protein-1
MA	methylamine
MAC	macroglobulin activated for cytokine binding
MAPK	mitogen-activated protein kinases
MASP	mannose-binding protein-associated serine protease
MBP	mannose-binding protein
MBL	mannose-binding lectin
MG	macroglobulin-like domain
MMP	matrix metalloproteinase
MT1-MMP	membrane-type 1 matrix metalloproteinase
NE	neutrophil elastase
NF-κB	nuclear factor kappa-light-chain-enhancer of activated B cells
NGF	nerve growth factor
NMDA	N-methyl-D-aspartate
OA	osteoarthritis
P3	proteinase 3
p75NTR	p75 neurotrophin receptor
PAK2	p21-activated protein kinase-2
PDGF	platelet-derived growth factor
PGE2	prostaglandin E2
PLCγ1	phospholipase Cγ1
PSA	prostate-specific antigen
PSD	post synaptic density protein
PTB	phosphotyrosine-binding
PTGS2	prostaglandin-endoperoxide synthase-2
RA	rheumatoid arthritis
RAP	receptor-associated protein
RBD	receptor binding domain
RhoA	Ras homolog family member A
Sh2	Src homology 2
SHP-2	Sh2-containing protein tyrosine phosphatase 2
ROS	reactive oxygen species
SMCs	smooth muscle cells
TED	thioester domain
TEP	thioester-containing protein
TGF	transforming growth factor
TNF	tumor necrosis factor
trk	tyrosine receptor kinase
tPA	tissue plasminogen activator
VEGF	vascular endothelial growth factor

## References

[1] Armstrong PB Quigley JP. Alpha2-Macroglobulin: An Evolutionarily Conserved Arm of the Innate Immune System. Dev Comp Immunol (1999) 23:375–90. doi: 10.1016/s0145-305x(99)00018-x 10426429

[2] Marrero A Duquerroy S Trapani S Goulas T Guevara T Andersen GR. The Crystal Structure of Human Alpha2-Macroglobulin Reveals a Unique Molecular Cage. Angew Chem Int Ed Engl (2012) 51:3340–4. doi: 10.1002/anie.201108015 22290936

[3] Nagasawa S Han BH Sugihara H Suzuki T. Studies on Alpha 2-Macroglobulin in Bovine Plasma. II. Interaction of Alpha-2-Macroglobulin and Trypsin. J Biochem (1970) 67:821–32. doi: 10.1093/oxfordjournals.jbchem.a129314 5448851

[4] Barrett AJ Starkey PM. The Interaction of Alpha 2-Macroglobulin With Proteinases. Characteristics and Specificity of the Reaction, and a Hypothesis Concerning its Molecular Mechanism. Biochem J (1973) 133:709–24. doi: 10.1042/bj1330709 PMC1177761 4201304

[5] Sottrup-Jensen L. Alpha-Macroglobulins: Structure, Shape, and Mechanism of Proteinase Complex Formation. J Biol Chem (1989) 264:11539–42. doi: 10.1016/S0021-9258(18)80094-1 2473064

[6] Sottrup-Jensen L Lønblad PB Stepanik TM Petersen TE Magnusson S Jörnvall H. Primary Structure of the 'Bait' Region for Proteinases in α2-Macroglobulin. Nature of the Complex. FEBS Lett (1981) 127:167–73. doi: 10.1016/0014-5793(81)80197-4 6165619

[7] Salvesen GS Barrett AJ. Covalent Binding of Proteinases in Their Reaction With Alpha-2-Macroglobulin. Biochem J (1980) 187:695–701. doi: 10.1042/bj1870695 6204637 PMC1162453

[8] Goulas T Garcia-Ferrer I Marrero A Marino-Puertas L Duquerroy S Gomis-Rüth FX. Structural and Functional Insight Into Pan-Endopeptidase Inhibition by α2-Macroglobulins. Biol Chem (2017) 398:975–94. doi: 10.1515/hsz-2016-0329 28253193

[9] Gonias SL Reynolds JA Pizzo SV. Physical Properties of Human Alpha 2-Macroglobulin Following Reaction With Methylamine and Trypsin. Biochim Biophys Acta (1982) 705:306–14. doi: 10.1016/0167-4838(82)90252-7 6181812

[10] Kristensen T Moestrup SK Gliemann J Bendtsen L Sand O Sottrup-Jensen L. Evidence That the Newly Cloned Low-Density-Lipoprotein Receptor Related Protein (LRP) is the Alpha 2-Macroglobulin Receptor. FEBS Lett (1990) 276:151–5. doi: 10.1016/0014-5793(90)80530-v 1702392

[11] Herz J Strickland DK. LRP: A Multifunctional Scavenger and Signaling Receptor. J Clin Invest (2001) 108:779–84. doi: 10.1172/JCI13992 PMC200939 11560943

[12] Misra UK Chu CT Gawdi G Pizzo SV. Evidence for a Second Alpha-2-Macroglobulin Receptor. J Biol Chem (1994) 269:12541–7. doi: 10.1016/S0021-9258(18)99909-6 7513689

[13] Misra UK Gonzalez-Gronow M Gawdi G Hart JP Johnson CE Pizzo SV. The Role of Grp78 in Alpha-2-Macroglobulin-Induced Signal Transduction. Evidence From RNA Interference That the Low Density Lipoprotein Receptor-Related Protein is Associated With, But Not Necessary for, GRP 78-Mediated Signal Transduction. J Biol Chem (2002) 277:42082–7. doi: 10.1074/jbc.M206174200 12194978

[14] Misra UK Chu CT Rubenstein DS Gawdi G Pizzo SV. Receptor-Recognized Alpha-2-Macroglobulin-Methylamine Elevates Intracellular Calcium, Inositol Phosphates and Cyclic AMP in Murine Peritoneal Macrophages. Biochem J (1993) 290:885–91. doi: 10.1042/bj2900885 PMC1132363 7681282

[15] Chu CT Pizzo SV. Receptor-Mediated Antigen Delivery Into Macrophages. Complexing Antigen to Alpha 2-Macroglobulin Enhances Presentation to T Cells. J Immunol (1993) 150:48–58. 7678035

[16] Bowers EV Horvath JJ Bond JE Cianciolo GJ Pizzo SV. Antigen Delivery by Alpha(2)-Macroglobulin Enhances the Cytotoxic T Lymphocyte Response. J Leukoc Biol (2009) 86:1259–68. doi: 10.1189/jlb.1008653 PMC2774884 19652028

[17] Huang JS Huang SS Deuel TF. Specific Covalent Binding of Platelet-Derived Growth Factor to Human Plasma Alpha 2-Macroglobulin. Proc Natl Acad Sci U S A (1984) 81:342–6. doi: 10.1073/pnas.81.2.342 PMC344672 6198647

[18] James K. Interactions Between Cytokines and Alpha 2-Macroglobulin. Immunol Today (1990) 11:163–6. doi: 10.1016/0167-5699(90)90165-6 1692465

[19] Cater JH Wilson MR Wyatt AR. Alpha-2-Macroglobulin, a Hypochlorite-Regulated Chaperone and Immune System Modulator. Oxid Med Cell Longev (2019) 2019:5410657. doi: 10.1155/2019/5410657 31428227 PMC6679887

[20] Sottrup-Jensen L Stepanik TM Kristensen T Lonblad PB Jones CM Wierzbicki DM. Common Evolutionary Origin of Alpha 2-Macroglobulin and Complement Components C3 and C4. Proc Natl Acad Sci U S A (1985) 82:9–13. doi: 10.1073/pnas.82.1.9 2578664 PMC396960

[21] Ricklin D Reis ES Mastellos DC Gros P Lambris JD. Complement Component C3 - The "Swiss Army Knife" of Innate Immunity and Host Defense. Immunol Rev (2016) 274:33–58. doi: 10.1111/imr.12500 27782325 PMC5427221

[22] Quigley JP Armstrong PB. Invertebrate Alpha 2-Macroglobulin: Structure-Function and the Ancient Thiol Ester Bond. Ann N Y Acad Sci (1994) 712:131–45. doi: 10.1111/j.1749-6632.1994.tb33568.x 7514851

[23] Shokal U Eleftherianos I. Evolution and Function of Thioester-Containing Proteins and the Complement System in the Innate Immune Response. Front Immunol (2017) 8:759. doi: 10.3389/fimmu.2017.00759 28706521 PMC5489563

[24] Janssen BJ Huizinga EG Raaijmakers HC Roos A Daha MR Nilsson-Ekdahl K. Structures of Complement Component C3 Provide Insights Into the Function and Evolution of Immunity. Nature (2005) 437:505–11. doi: 10.1038/nature04005 16177781

[25] Garcia-Ferrer I Marrero A Gomis-Ruth FX Goulas T. Alpha-2-Macroglobulins: Structure and Function. Subcell Biochem (2017) 83:149–83. doi: 10.1007/978-3-319-46503-6_6 28271476

[26] Enghild JJ Thogersen IB Salvesen G Fey GH Figler NL Gonias SL. Alpha-Macroglobulin From *Limulus Polyphemus* Exhibits Proteinase Inhibitory Activity and Participates in a Hemolytic System. Biochemistry (1990) 29:10070–80. doi: 10.1021/bi00495a009 1703001

[27] Armstrong PB Melchior R Swarnakar S Quigley JP. Alpha-2-Macroglobulin Does Not Function as a C3 Homologue in the Plasma Hemolytic System of the American Horseshoe Crab, *Limulus* . Mol Immunol (1998) 35:47–53. doi: 10.1016/s0161-5890(98)00007-8 9683263

[28] Salvesen GS Sayers CA Barrett AJ. Further Characterization of the Covalent Linking Reaction of Alpha 2-Macroglobulin. Biochem J (1981) 195:453–61. doi: 10.1042/bj1950453 PMC1162909 6172116

[29] Dodds AW Law SK. The Phylogeny and Evolution of the Thioester Bond-Containing Proteins C3, C4 and Alpha 2-Macroglobulin. Immunol Rev (1998) 166:15–26. doi: 10.1111/j.1600-065x.1998.tb01249.x 9914899

[30] Chu CT Pizzo SV. Alpha 2-Macroglobulin, Complement, and Biologic Defense: Antigens, Growth Factors, Microbial Proteases, and Receptor Ligation. Lab Invest (1994) 71:792–812. 7528831

[31] Terai I Kobayashi K Matsushita M Fujita T Matsuno K. Alpha 2-Macroglobulin Binds to and Inhibits Mannose-Binding Protein-Associated Serine Protease. Int Immunol (1995) 7:1579–84. doi: 10.1093/intimm/7.10.1579 8562502

[32] Ambrus G Gal P Kojima M Szilagyi K Balczer J Antal J. Natural Substrates and Inhibitors of Mannan-Binding Lectin-Associated Serine Protease-1 and -2: A Study on Recombinant Catalytic Fragments. J Immunol (2003) 170:1374–82. doi: 10.4049/jimmunol.170.3.1374 12538697

[33] Parej K Dobo J Zavodszky P Gal P. The Control of the Complement Lectin Pathway Activation Revisited: Both C1-Inhibitor and Antithrombin are Likely Physiological Inhibitors, While Alpha-2-Macroglobulin is Not. Mol Immunol (2013) 54:415–22. doi: 10.1016/j.molimm.2013.01.009 23399388

[34] Storgaard P Holm Nielsen E Skriver E Andersen O Svehag SE. Mannan-Binding Protein Forms Complexes With Alpha-2-Macroglobulin. A Protein Model for the Interaction. Scand J Immunol (1995) 42:373–80. doi: 10.1111/j.1365-3083.1995.tb03670.x 7544912

[35] Arnold JN Wallis R Willis AC Harvey DJ Royle L Dwek RA. Interaction of Mannan Binding Lectin With Alpha-2-Macroglobulin *via* Exposed Oligomannose Glycans: A Conserved Feature of the Thiol Ester Protein Family? J Biol Chem (2006) 281:6955–63. doi: 10.1074/jbc.M511432200 16407218

[36] Naseraldeen N Michelis R Barhoum M Chezar J Tadmor T Aviv A. The Role of Alpha-2-Macroglobulin in IgG-Aggregation and Chronic Activation of the Complement System in Patients With Chronic Lymphocytic Leukemia. Front Immunol (2020) 11:603569. doi: 10.3389/fimmu.2020.603569 33643290 PMC7905172

[37] McDaniel MC Laudico R Papermaster BW. Association of Macrophage-Activation Factor From a Human Cultured Lymphoid Cell Line With Albumin and Alpha-2-Macroglobulin. Clin Immunol Immunopathol (1976) 5:91–104. doi: 10.1016/0090-1229(76)90153-7 57021

[38] Ronne H Anundi H Rask L Peterson PA. Nerve Growth Factor Binds to Serum Alpha-2-Macroglobulin. Biochem Biophys Res Commun (1979) 87:330–6. doi: 10.1016/0006-291x(79)91683-8 88223

[39] Asplin IR Wu SM Mathew S Bhattacharjee G Pizzo SV. Differential Regulation of the Fibroblast Growth Factor (FGF) Family by Alpha(2)-Macroglobulin: Evidence for Selective Modulation of FGF-2-Induced Angiogenesis. Blood (2001) 97:3450–7. doi: 10.1182/blood.v97.11.3450 11369636

[40] Crookston KP Webb DJ Wolf BB Gonias SL. Classification of Alpha-2-Macroglobulin-Cytokine Interactions Based on Affinity of Noncovalent Association in Solution Under Apparent Equilibrium Conditions. J Biol Chem (1994) 269:1533–40. doi: 10.1016/S0021-9258(17)42289-7 7507109

[41] Dennis PA Saksela O Harpel P Rifkin DB. Alpha 2-Macroglobulin is a Binding Protein for Basic Fibroblast Growth Factor. J Biol Chem (1989) 264:7210–6. doi: 10.1016/S0021-9258(18)83222-7 2468667

[42] James K van den Haan J Lens S Farmer K. Preliminary Studies on the Interaction of TNF Alpha and IFN Gamma With Alpha 2-Macroglobulin. Immunol Lett (1992) 32:49–57. doi: 10.1016/0165-2478(92)90198-w 1379978

[43] Borth W Luger TA. Identification of Alpha 2-Macroglobulin as a Cytokine Binding Plasma Protein. Binding of Interleukin-1 Beta to "F" Alpha 2-Macroglobulin. J Biol Chem (1989) 264:5818–25. doi: 10.1016/S0021-9258(18)83623-7 2466831

[44] Arandjelovic S Dragojlovic N Li X Myers RR Campana WM Gonias SL. A Derivative of the Plasma Protease Inhibitor Alpha(2)-Macroglobulin Regulates the Response to Peripheral Nerve Injury. J Neurochem (2007) 103:694–705. doi: 10.1111/j.1471-4159.2007.04800.x 17725582

[45] Borth W Scheer B Urbansky A Luger TA Sottrup-Jensen L. Binding of IL-1 Beta to Alpha-Macroglobulins and Release by Thioredoxin. J Immunol (1990) 145:3747–54. 1700994

[46] Wu SM Patel DD Pizzo SV. Oxidized Alpha2-Macroglobulin (Alpha2m) Differentially Regulates Receptor Binding by Cytokines/Growth Factors: Implications for Tissue Injury and Repair Mechanisms in Inflammation. J Immunol (1998) 161:4356–65. 9780213

[47] Matsuda T Hirano T Nagasawa S Kishimoto T. Identification of Alpha 2-Macroglobulin as a Carrier Protein for IL-6. J Immunol (1989) 142:148–52. 2462586

[48] Kurdowska A Carr FK Stevens MD Baughman RP Martin TR. Studies on the Interaction of IL-8 With Human Plasma Alpha 2-Macroglobulin: Evidence for the Presence of IL-8 Complexed to Alpha 2-Macroglobulin in Lung Fluids of Patients With Adult Respiratory Distress Syndrome. J Immunol (1997) 158:1930–40. 9029135

[49] Gonias SL Carmichael A Mettenburg JM Roadcap DW Irvin WP Webb DJ. Identical or Overlapping Sequences in the Primary Structure of Human Alpha(2)-Macroglobulin are Responsible for the Binding of Nerve Growth Factor-Beta, Platelet-Derived Growth Factor-BB, and Transforming Growth Factor-Beta. J Biol Chem (2000) 275:5826–31. doi: 10.1074/jbc.275.8.5826 10681572

[50] Bonner JC Goodell AL Lasky JA Hoffman MR. Reversible Binding of Platelet-Derived Growth Factor-AA, -AB, and -BB Isoforms to a Similar Site on the "Slow" and "Fast" Conformations of Alpha 2-Macroglobulin. J Biol Chem (1992) 267:12837–44. doi: 10.1016/S0021-9258(18)42352-6 1377675

[51] Webb DJ Roadcap DW Dhakephalkar A Gonias SL. A 16-Amino Acid Peptide From Human Alpha2-Macroglobulin Binds Transforming Growth Factor-Beta and Platelet-Derived Growth Factor-BB. Protein Sci (2000) 9:1986–92. doi: 10.1110/ps.9.10.1986 PMC2144455 11106172

[52] Arandjelovic S Van Sant CL Gonias SL. Limited Mutations in Full-Length Tetrameric Human Alpha2-Macroglobulin Abrogate Binding of Platelet-Derived Growth Factor-BB and Transforming Growth Factor-Beta1. J Biol Chem (2006) 281:17061–8. doi: 10.1074/jbc.M602217200 16641085

[53] Raines EW Bowen-Pope DF Ross R. Plasma Binding Proteins for Platelet-Derived Growth Factor That Inhibit its Binding to Cell-Surface Receptors. Proc Natl Acad Sci U S A (1984) 81:3424–8. doi: 10.1073/pnas.81.11.3424 PMC345520 6203121

[54] Crookston KP Webb DJ Lamarre J Gonias SL. Binding of Platelet-Derived Growth Factor-BB and Transforming Growth Factor-Beta 1 to Alpha 2-Macroglobulin *In Vitro* and *In Vivo*: Comparison of Receptor-Recognized and Non-Recognized Alpha 2-Macroglobulin Conformations. Biochem J (1993) 293:443–50. doi: 10.1042/bj2930443 PMC1134380 7688216

[55] Huang SS O'Grady P Huang JS. Human Transforming Growth Factor Beta.Alpha 2-Macroglobulin Complex Is a Latent Form of Transforming Growth Factor Beta. J Biol Chem (1988) 263:1535–41. doi: 10.1016/S0021-9258(19)57337-9 2447091

[56] Webb DJ Wen J Karns LR Kurilla MG Gonias SL. Localization of the Binding Site for Transforming Growth Factor-Beta in Human Alpha2-Macroglobulin to a 20-kDa Peptide That Also Contains the Bait Region. J Biol Chem (1998) 273:13339–46. doi: 10.1074/jbc.273.21.13339 9582381

[57] O'Connor-McCourt MD Wakefield LM. Latent Transforming Growth Factor-Beta in Serum. A Specific Complex With Alpha 2-Macroglobulin. J Biol Chem (1987) 262:14090–9. doi: 10.1016/S0021-9258(18)47909-4 2443501

[58] LaMarre J Hayes MA Wollenberg GK Hussaini I Hall SW Gonias SL. An Alpha 2-Macroglobulin Receptor-Dependent Mechanism for the Plasma Clearance of Transforming Growth Factor-Beta 1 in Mice. J Clin Invest (1991) 87:39–44. doi: 10.1172/JCI114998 1702100 PMC294986

[59] Hall SW LaMarre J Marshall LB Hayes MA Gonias SL. Binding of Transforming Growth Factor-Beta 1 to Methylamine-Modified Alpha 2-Macroglobulin and to Binary and Ternary Alpha 2-Macroglobulin-Proteinase Complexes. Biochem J (1992) 281:569–75. doi: 10.1042/bj2810569 PMC1130723 1371050

[60] Stouffer GA LaMarre J Gonias SL Owens GK. Activated Alpha 2-Macroglobulin and Transforming Growth Factor-Beta 1 Induce a Synergistic Smooth Muscle Cell Proliferative Response. J Biol Chem (1993) 268:18340–4. doi: 10.1016/S0021-9258(17)46850-5 7688745

[61] Wollenberg GK LaMarre J Rosendal S Gonias SL Hayes MA. Binding of Tumor Necrosis Factor Alpha to Activated Forms of Human Plasma Alpha 2 Macroglobulin. Am J Pathol (1991) 138:265–72. PMC1886187 1704186

[62] Bhattacharjee G Asplin IR Wu SM Gawdi G Pizzo SV. The Conformation-Dependent Interaction of Alpha 2-Macroglobulin With Vascular Endothelial Growth Factor. A Novel Mechanism of Alpha 2-Macroglobulin/Growth Factor Binding. J Biol Chem (2000) 275:26806–11. doi: 10.1074/jbc.M000156200 10862607

[63] Soker S Svahn CM Neufeld G. Vascular Endothelial Growth Factor Is Inactivated by Binding to Alpha 2-Macroglobulin and the Binding Is Inhibited by Heparin. J Biol Chem (1993) 268:7685–91. doi: 10.1016/S0021-9258(18)53011-8 7681826

[64] Danielpour D Sporn MB. Differential Inhibition of Transforming Growth Factor Beta 1 and Beta 2 Activity by Alpha 2-Macroglobulin. J Biol Chem (1990) 265:6973–7. doi: 10.1016/S0021-9258(19)39246-4 1691181

[65] Philip A O'Connor-McCourt MD. Interaction of Transforming Growth Factor-Beta 1 With Alpha 2-Macroglobulin. Role in Transforming Growth Factor-Beta 1 Clearance. J Biol Chem (1991) 266:22290–6. doi: 10.1016/S0021-9258(18)54568-3 1718991

[66] Webb DJ Gonias SL. Chemical Modification of Alpha2-Macroglobulin to Generate Derivatives That Bind Transforming Growth Factor-Beta With Increased Affinity. FEBS Lett (1997) 410:249–53. doi: 10.1016/s0014-5793(97)00598-x 9237639

[67] Webb DJ Gonias SL. A Modified Human Alpha 2-Macroglobulin Derivative That Binds Tumor Necrosis Factor-Alpha and Interleukin-1 Beta With High Affinity *In Vitro* and Reverses Lipopolysaccharide Toxicity *In Vivo* in Mice. Lab Invest (1998) 78:939–48. 9714181

[68] Gourine AV Gourine VN Tesfaigzi Y Caluwaerts N Van Leuven F Kluger MJ. Role of Alpha(2)-Macroglobulin in Fever and Cytokine Responses Induced by Lipopolysaccharide in Mice. Am J Physiol Regul Integr Comp Physiol (2002) 283:R218–26. doi: 10.1152/ajpregu.00746.2001 12069948

[69] Herz J Hamann U Rogne S Myklebost O Gausepohl H Stanley KK. Surface Location and High Affinity for Calcium of a 500-Kd Liver Membrane Protein Closely Related to the LDL-Receptor Suggest a Physiological Role as Lipoprotein Receptor. EMBO J (1988) 7:4119–27. doi: 10.1002/j.1460-2075.1988.tb03306.x PMC455121 3266596

[70] Strickland DK Ashcom JD Williams S Battey F Behre E McTigue K. Primary Structure of Alpha 2-Macroglobulin Receptor-Associated Protein. Human Homologue of a Heymann Nephritis Antigen. J Biol Chem (1991) 266:13364–9. doi: 10.1016/S0021-9258(18)98848-4 1712782

[71] Williams SE Ashcom JD Argraves WS Strickland DK. A Novel Mechanism for Controlling the Activity of Alpha 2-Macroglobulin Receptor/Low Density Lipoprotein Receptor-Related Protein. Multiple Regulatory Sites for 39-kDa Receptor-Associated Protein. J Biol Chem (1992) 267:9035–40. doi: 10.1016/S0021-9258(19)50384-2 1374383

[72] Daly NL Scanlon MJ Djordjevic JT Kroon PA Smith R. Three-Dimensional Structure of a Cysteine-Rich Repeat From the Low-Density Lipoprotein Receptor. Proc Natl Acad Sci U S A (1995) 92:6334–8. doi: 10.1073/pnas.92.14.6334 PMC41512 7603991

[73] Emonard H Theret L Bennasroune AH Dedieu S. Regulation of LRP-1 Expression: Make the Point. Pathol Biol (Paris) (2014) 62:84–90. doi: 10.1016/j.patbio.2014.02.002 24661974

[74] Bres EE Faissner A. Low Density Receptor-Related Protein 1 Interactions With the Extracellular Matrix: More Than Meets the Eye. Front Cell Dev Biol (2019) 7:31. doi: 10.3389/fcell.2019.00031 30931303 PMC6428713

[75] Li Y Marzolo MP van Kerkhof P Strous GJ Bu G. The YXXL Motif, But Not the Two NPXY Motifs, Serves as the Dominant Endocytosis Signal for Low Density Lipoprotein Receptor-Related Protein. J Biol Chem (2000) 275:17187–94. doi: 10.1074/jbc.M000490200 10747918

[76] Willingham MC Maxfield FR Pastan I. Receptor-Mediated Endocytosis of Alpha 2-Macroglobulin in Cultured Fibroblasts. J Histochem Cytochem (1980) 28:818–23. doi: 10.1177/28.8.6160180 6160180

[77] Holtet TL Nielsen KL Etzerodt M Moestrup SK Gliemann J Sottrup-Jensen L. Receptor-Binding Domain of Human Alpha 2-Macroglobulin. Expression, Folding and Biochemical Characterization of a High-Affinity Recombinant Derivative. FEBS Lett (1994) 344:242–6. doi: 10.1016/0014-5793(94)00349-1 7514545

[78] Howard GC Yamaguchi Y Misra UK Gawdi G Nelsen A DeCamp DL. Selective Mutations in Cloned and Expressed Alpha-Macroglobulin Receptor Binding Fragment Alter Binding to Either the Alpha2-Macroglobulin Signaling Receptor or the Low Density Lipoprotein Receptor-Related Protein/Alpha2-Macroglobulin Receptor. J Biol Chem (1996) 271:14105–11. doi: 10.1074/jbc.271.24.14105 8662881

[79] Nielsen KL Holtet TL Etzerodt M Moestrup SK Gliemann J Sottrup-Jensen L. Identification of Residues in Alpha-Macroglobulins Important for Binding to the Alpha2-Macroglobulin Receptor/Low Density Lipoprotein Receptor-Related Protein. J Biol Chem (1996) 271:12909–12. doi: 10.1074/jbc.271.22.12909 8662686

[80] Arandjelovic S Hall BD Gonias SL. Mutation of Lysine 1370 in Full-Length Human Alpha2-Macroglobulin Blocks Binding to the Low Density Lipoprotein Receptor-Related Protein-1. Arch Biochem Biophys (2005) 438:29–35. doi: 10.1016/j.abb.2005.03.019 15910735

[81] Moestrup SK Gliemann J. Analysis of Ligand Recognition by the Purified Alpha 2-Macroglobulin Receptor (Low Density Lipoprotein Receptor-Related Protein). Evidence That High Affinity of Alpha 2-Macroglobulin-Proteinase Complex is Achieved by Binding to Adjacent Receptors. J Biol Chem (1991) 266:14011–7. doi: 10.1016/S0021-9258(18)92803-6 1713212

[82] Delain E Barray M Pochon F Gliemann J Moestrup SK. Electron Microscopic Visualization of the Human Alpha 2-Macroglobulin Receptor and its Interaction With Alpha 2-Macroglobulin/Chymotrypsin Complex. Ann N Y Acad Sci (1994) 737:202–11. doi: 10.1111/j.1749-6632.1994.tb44313.x 7524397

[83] Mikhailenko I Battey FD Migliorini M Ruiz JF Argraves K Moayeri M. Recognition of Alpha 2-Macroglobulin by the Low Density Lipoprotein Receptor-Related Protein Requires the Cooperation of Two Ligand Binding Cluster Regions. J Biol Chem (2001) 276:39484–91. doi: 10.1074/jbc.M104382200 11507091

[84] Gotthardt M Trommsdorff M Nevitt MF Shelton J Richardson JA Stockinger W. Interactions of the Low Density Lipoprotein Receptor Gene Family With Cytosolic Adaptor and Scaffold Proteins Suggest Diverse Biological Functions in Cellular Communication and Signal Transduction. J Biol Chem (2000) 275:25616–24. doi: 10.1074/jbc.M000955200 10827173

[85] Betts GN van der Geer P Komives EA. Structural and Functional Consequences of Tyrosine Phosphorylation in the LRP1 Cytoplasmic Domain. J Biol Chem (2008) 283:15656–64. doi: 10.1074/jbc.M709514200 PMC2414285 18381291

[86] Mantuano E Lam MS Gonias SL. LRP1 Assembles Unique Co-Receptor Systems to Initiate Cell Signaling in Response to Tissue-Type Plasminogen Activator and Myelin-Associated Glycoprotein. J Biol Chem (2013) 288:34009–18. doi: 10.1074/jbc.M113.509133 PMC3837140 24129569

[87] Uhlén M Fagerberg L Hallström BM Lindskog C Oksvold P Mardinoglu A. Proteomics. Tissue-Based Map of the Human Proteome. Science (2015) 347:1260419. doi: 10.1126/science.1260419 25613900

[88] Moestrup SK Gliemann J Pallesen G. Distribution of the Alpha 2-Macroglobulin Receptor/Low Density Lipoprotein Receptor-Related Protein in Human Tissues. Cell Tissue Res (1992) 269:375–82. doi: 10.1007/BF00353892 1423505

[89] Yamamoto K Santamaria S Botkjaer KA Dudhia J Troeberg L Itoh Y. Inhibition of Shedding of Low-Density Lipoprotein Receptor-Related Protein 1 Reverses Cartilage Matrix Degradation in Osteoarthritis. Arthritis Rheumatol (2017) 69:1246–56. doi: 10.1002/art.40080 PMC5449214 28235248

[90] Gorovoy M Gaultier A Campana WM Firestein GS Gonias SL. Inflammatory Mediators Promote Production of Shed LRP1/CD91, Which Regulates Cell Signaling and Cytokine Expression by Macrophages. J Leukoc Biol (2010) 88:769–78. doi: 10.1189/jlb.0410220 PMC2974427 20610799

[91] Gaultier A Arandjelovic S Niessen S Overton CD Linton MF Fazio S. Regulation of Tumor Necrosis Factor Receptor-1 and the IKK-NF-kappaB Pathway by LDL Receptor-Related Protein Explains the Antiinflammatory Activity of This Receptor. Blood (2008) 111:5316–25. doi: 10.1182/blood-2007-12-127613 PMC2396725 18369152

[92] Misra UK Chu CT Gawdi G Pizzo SV. The Relationship Between Low Density Lipoprotein-Related Protein/Alpha 2-Macroglobulin (Alpha 2M) Receptors and the Newly Described Alpha 2M Signaling Receptor. J Biol Chem (1994) 269:18303–6. doi: 10.1016/S0021-9258(17)32305-0 7518427

[93] Stolz A Wolf DH. Endoplasmic Reticulum Associated Protein Degradation: A Chaperone Assisted Journey to Hell. Biochim Biophys Acta (2010) 1803:694–705. doi: 10.1016/j.bbamcr.2010.02.005 20219571

[94] Lee AS. The Glucose-Regulated Proteins: Stress Induction and Clinical Applications. Trends Biochem Sci (2001) 26:504–10. doi: 10.1016/s0968-0004(01)01908-9 11504627

[95] Sukata T Uwagawa S Ozaki K Sumida K Kushida M Kakehashi A. Characteristic Upregulation of Glucose-Regulated Protein 78 in an Early Lesion Negative for Hitherto Established Cytochemical Markers in Rat Hepatocarcinogenesis. J Toxicol Pathol (2009) 22:281–8. doi: 10.1293/tox.22.281 PMC3234602 22272003

[96] Farshbaf M Khosroushahi AY Mojarad-Jabali S Zarebkohan A Valizadeh H Walker PR. Cell Surface GRP78: An Emerging Imaging Marker and Therapeutic Target for Cancer. J Control Release (2020) 328:932–41. doi: 10.1016/j.jconrel.2020.10.055 33129921

[97] Misra UK Gonzalez-Gronow M Gawdi G Pizzo SV. The Role of MTJ-1 in Cell Surface Translocation of GRP78, a Receptor for Alpha 2-Macroglobulin-Dependent Signaling. J Immunol (2005) 174:2092–7. doi: 10.4049/jimmunol.174.4.2092 15699139

[98] Piette BL Alerasool N Lin ZY Lacoste J Lam MHY Qian WW. Comprehensive Interactome Profiling of the Human Hsp70 Network Highlights Functional Differentiation of J Domains. Mol Cell (2021) 81:2549–65.e8. doi: 10.1016/j.molcel.2021.04.012 33957083

[99] Misra UK Wang F Pizzo SV. Transcription Factor TFII-I Causes Transcriptional Upregulation of GRP78 Synthesis in Prostate Cancer Cells. J Cell Biochem (2009) 106:381–9. doi: 10.1002/jcb.22016 19097122

[100] Misra UK Payne S Pizzo SV. Ligation of Prostate Cancer Cell Surface GRP78 Activates a Proproliferative and Antiapoptotic Feedback Loop: A Role for Secreted Prostate-Specific Antigen. J Biol Chem (2011) 286:1248–59. doi: 10.1074/jbc.M110.129767 PMC3020732 21056970

[101] Salvesen G Virca GD Travis J. Interaction of Alpha 2-Macroglobulin With Neutrophil and Plasma Proteinases. Ann N Y Acad Sci (1983) 421:316–26. doi: 10.1111/j.1749-6632.1983.tb18120.x 6202199

[102] Campbell EJ Senior RM McDonald JA Cox DL. Proteolysis by Neutrophils. Relative Importance of Cell-Substrate Contact and Oxidative Inactivation of Proteinase Inhibitors *In Vitro* . J Clin Invest (1982) 70:845–52. doi: 10.1172/jci110681 PMC370293 6181097

[103] Stone PJ Calore JD Franzblau C. Release of Human Neutrophil Elastase From Alpha 2-Macroglobulin Complexes Containing Human Neutrophil Elastase. Ann N Y Acad Sci (1983) 421:398–400. doi: 10.1111/j.1749-6632.1983.tb18132.x 6202211

[104] Wewers MD Herzyk DJ Gadek JE. Alveolar Fluid Neutrophil Elastase Activity in the Adult Respiratory Distress Syndrome is Complexed to Alpha-2-Macroglobulin. J Clin Invest (1988) 82:1260–7. doi: 10.1172/JCI113724 PMC442677 2459160

[105] Rao NV Wehner NG Marshall BC Gray WR Gray BH Hoidal JR. Characterization of Proteinase-3 (PR-3), a Neutrophil Serine Proteinase. Structural and Functional Properties. J Biol Chem (1991) 266:9540–8. doi: 10.1016/S0021-9258(18)92854-1 2033050

[106] Nagase H Itoh Y Binner S. Interaction of Alpha 2-Macroglobulin With Matrix Metalloproteinases and its Use for Identification of Their Active Forms. Ann N Y Acad Sci (1994) 732:294–302. doi: 10.1111/j.1749-6632.1994.tb24744.x 7526760

[107] Serifova X Ugarte-Berzal E Opdenakker G Vandooren J. Homotrimeric MMP-9 is an Active Hitchhiker on Alpha-2-Macroglobulin Partially Escaping Protease Inhibition and Internalization Through LRP-1. Cell Mol Life Sci (2019) 77:3013–26. doi: 10.1007/s00018-019-03338-4 PMC11104829 31642940

[108] Reddy VY Pizzo SV Weiss SJ. Functional Inactivation and Structural Disruption of Human Alpha 2-Macroglobulin by Neutrophils and Eosinophils. J Biol Chem (1989) 264:13801–9. doi: 10.1016/S0021-9258(18)80072-2 2474536

[109] Siddiqui T Zia MK Ali SS Ahsan H Khan FH. Insight Into the Interactions of Proteinase Inhibitor- Alpha-2-Macroglobulin With Hypochlorite. Int J Biol Macromol (2018) 117:401–6. doi: 10.1016/j.ijbiomac.2018.05.112 29778882

[110] Reddy VY Desorchers PE Pizzo SV Gonias SL Sahakian JA Levine RL. Oxidative Dissociation of Human Alpha 2-Macroglobulin Tetramers Into Dysfunctional Dimers. J Biol Chem (1994) 269:4683–91. doi: 10.1016/S0021-9258(17)41830-8 7508448

[111] Wu SM Pizzo SV. Alpha(2)-Macroglobulin From Rheumatoid Arthritis Synovial Fluid: Functional Analysis Defines a Role for Oxidation in Inflammation. Arch Biochem Biophys (2001) 391:119–26. doi: 10.1006/abbi.2001.2408 11414692

[112] Wyatt AR Kumita JR Mifsud RW Gooden CA Wilson MR Dobson CM. Hypochlorite-Induced Structural Modifications Enhance the Chaperone Activity of Human Alpha2-Macroglobulin. Proc Natl Acad Sci U S A (2014) 111:E2081–90. doi: 10.1073/pnas.1403379111 PMC4034205 24799681

[113] French K Yerbury JJ Wilson MR. Protease Activation of Alpha2-Macroglobulin Modulates a Chaperone-Like Action With Broad Specificity. Biochemistry (2008) 47:1176–85. doi: 10.1021/bi701976f 18171086

[114] Wyatt AR Constantinescu P Ecroyd H Dobson CM Wilson MR Kumita JR. Protease-Activated Alpha-2-Macroglobulin can Inhibit Amyloid Formation *via* Two Distinct Mechanisms. FEBS Lett (2013) 587:398–403. doi: 10.1016/j.febslet.2013.01.020 23353684 PMC3581772

[115] Dalli J Norling LV Montero-Melendez T Federici Canova D Lashin H Pavlov AM. Microparticle Alpha-2-Macroglobulin Enhances Pro-Resolving Responses and Promotes Survival in Sepsis. EMBO Mol Med (2014) 6:27–42. doi: 10.1002/emmm.201303503 24357647 PMC3936490

[116] Dalli J Montero-Melendez T Norling LV Yin X Hinds C Haskard D. Heterogeneity in Neutrophil Microparticles Reveals Distinct Proteome and Functional Properties. Mol Cell Proteomics (2013) 12:2205–19. doi: 10.1074/mcp.M113.028589 PMC3734580 23660474

[117] Federici Canova D Pavlov AM Norling LV Gobbetti T Brunelleschi S Le Fauder P. Alpha-2-Macroglobulin Loaded Microcapsules Enhance Human Leukocyte Functions and Innate Immune Response. J Control Release (2015) 217:284–92. doi: 10.1016/j.jconrel.2015.09.021 PMC4649706 26385167

[118] Czarnetzki BM Schulz W. Role of Purified Serum Components in Polymorphonuclear Leukocyte Chemotaxis. Int Arch Allergy Appl Immunol (1980) 61:424–30. doi: 10.1159/000232470 6154015

[119] Forrester JV Wilkinson PC Lackie JM. Effect of Modified Alpha 2macroglobulin on Leucocyte Locomotion and Chemotaxis. Immunology (1983) 50:251–9. PMC1454339 6194105

[120] Chhatwal GS Albohn G Blobel H. Novel Complex Formed Between a Nonproteolytic Cell Wall Protein of Group A *Streptococci* and Alpha 2-Macroglobulin. J Bacteriol (1987) 169:3691–5. doi: 10.1128/jb.169.8.3691-3695.1987 PMC212452 2440851

[121] Sjobring U Trojnar J Grubb A Akerstrom B Bjorck L. Ig-Binding Bacterial Proteins Also Bind Proteinase Inhibitors. J Immunol (1989) 143:2948–54. 2478629

[122] Muller HP Rantamaki LK. Binding of Native Alpha 2-Macroglobulin to Human Group G *Streptococci* . Infect Immun (1995) 63:2833–9. doi: 10.1128/iai.63.8.2833-2839.1995 PMC173384 7542633

[123] Rasmussen M Muller HP Bjorck L. Protein GRAB of *Streptococcus Pyogenes* Regulates Proteolysis at the Bacterial Surface by Binding Alpha2-Macroglobulin. J Biol Chem (1999) 274:15336–44. doi: 10.1074/jbc.274.22.15336 10336419

[124] Valentin-Weigand P Traore MY Blobel H Chhatwal GS. Role of Alpha 2-Macroglobulin in Phagocytosis of Group A and C *Streptococci* . FEMS Microbiol Lett (1990) 58:321–4. doi: 10.1111/j.1574-6968.1990.tb13997.x 1699839

[125] He W Heinz A Jahn D Hiller K. Complexity of Macrophage Metabolism in Infection. Curr Opin Biotechnol (2021) 68:231–9. doi: 10.1016/j.copbio.2021.01.020 33610128

[126] White R Janoff A Godfrey HP. Secretion of Alpha-2-Macroglobulin by Human Alveolar Macrophages. Lung (1980) 158:9–14. doi: 10.1007/BF02713697 6157057

[127] Munck Petersen C Ejlersen E Wendelboe Hansen P Gliemann J. Binding of Alpha-2-Macroglobulin Trypsin Complex to Human Monocytes in Culture. Scand J Clin Lab Invest (1987) 47:55–61. doi: 10.3109/00365518709168870 2437644

[128] LaMarre J Wolf BB Kittler EL Quesenberry PJ Gonias SL. Regulation of Macrophage Alpha 2-Macroglobulin Receptor/Low Density Lipoprotein Receptor-Related Protein by Lipopolysaccharide and Interferon-Gamma. J Clin Invest (1993) 91:1219–24. doi: 10.1172/JCI116283 PMC288080 7680664

[129] Moestrup SK Kaltoft K Petersen CM Pedersen S Gliemann J Christensen EI. Immunocytochemical Identification of the Human Alpha 2-Macroglobulin Receptor in Monocytes and Fibroblasts: Monoclonal Antibodies Define the Receptor as a Monocyte Differentiation Antigen. Exp Cell Res (1990) 190:195–203. doi: 10.1016/0014-4827(90)90185-d 2209723

[130] Misra UK Gawdi G Pizzo SV. Ligation of the Alpha 2-Macroglobulin Signalling Receptor on Macrophages Induces Protein Phosphorylation and an Increase in Cytosolic pH. Biochem J (1995) 309:151–8. doi: 10.1042/bj3090151 PMC1135813 7542445

[131] Debanne MT Bell R Dolovich J. Uptake of Proteinase-Alpha-Macroglobulin Complexes by Macrophages. Biochim Biophys Acta (1975) 411:295–304. doi: 10.1016/0304-4165(75)90309-8 1201282

[132] Araujo-Jorge TC de Meirelles Mde N Isaac L. *Trypanosoma Cruzi*: Killing and Enhanced Uptake by Resident Peritoneal Macrophages Treated With Alpha-2-Macroglobulin. Parasitol Res (1990) 76:545–52. doi: 10.1007/BF00932558 1699221

[133] Ganter U Bauer J Schulz-Huotari C Gebicke-Haerter PJ Beeser H Gerok W. Repression of Alpha 2-Macroglobulin and Stimulation of Alpha 1-Proteinase Inhibitor Synthesis in Human Mononuclear Phagocytes by Endotoxin. Eur J Biochem (1987) 169:13–20. doi: 10.1111/j.1432-1033.1987.tb13574.x 2445565

[134] Bonacci GR Caceres LC Sanchez MC Chiabrando GA. Activated Alpha(2)-Macroglobulin Induces Cell Proliferation and Mitogen-Activated Protein Kinase Activation by LRP-1 in the J774 Macrophage-Derived Cell Line. Arch Biochem Biophys (2007) 460:100–6. doi: 10.1016/j.abb.2007.01.004 17288987

[135] Marzolo MP von Bernhardi R Bu G Inestrosa NC. Expression of Alpha(2)-Macroglobulin Receptor/Low Density Lipoprotein Receptor-Related Protein (LRP) in Rat Microglial Cells. J Neurosci Res (2000) 60:401–11. doi: 10.1002/(SICI)1097-4547(20000501)60:3<401::AID-JNR15>3.0.CO;2-L 10797543

[136] Misra UK Pizzo SV. Ligation of Alpha 2M Receptors With Alpha 2M-Methylamine Stimulates the Activities of Phospholipase C, Phospholipase A2, and Protein Kinase C in Murine Peritoneal Macrophages. Ann N Y Acad Sci (1994) 737:486–9. doi: 10.1111/j.1749-6632.1994.tb44347.x 7524426

[137] Misra UK Pizzo SV. Regulation of Cytosolic Phospholipase A2 Activity in Macrophages Stimulated With Receptor-Recognized Forms of Alpha 2-Macroglobulin: Role in Mitogenesis and Cell Proliferation. J Biol Chem (2002) 277:4069–78. doi: 10.1074/jbc.M109764200 11733496

[138] Misra UK Pizzo SV. Cytosolic Phospholipase A(2) Activity Associated With Nuclei is Not Inhibited by Arachidonyl Trifluoromethyl Ketone in Macrophages Stimulated With Receptor-Recognized Forms of Alpha(2)-Macroglobulin. Arch Biochem Biophys (2000) 379:153–60. doi: 10.1006/abbi.2000.1878 10864453

[139] Misra UK Akabani G Pizzo SV. The Role of cAMP-Dependent Signaling in Receptor-Recognized Forms of Alpha 2-Macroglobulin-Induced Cellular Proliferation. J Biol Chem (2002) 277:36509–20. doi: 10.1074/jbc.M203543200 12114513

[140] Misra UK Pizzo SV. Ligation of the Alpha2m Signaling Receptor With Receptor-Recognized Forms of Alpha2-Macroglobulin Initiates Protein and DNA Synthesis in Macrophages. The Effect of Intracellular Calcium. Biochim Biophys Acta (1998) 1401:121–8. doi: 10.1016/s0167-4889(97)00123-7 9459492

[141] Misra UK Pizzo SV. Ligation of the Alpha 2-Macroglobulin Signaling Receptor on Macrophages Induces Synthesis of Platelet Activating Factor. J Cell Biochem (1996) 61:39–47. doi: 10.1002/(SICI)1097-4644(19960401)61:1<39::AID-JCB6>3.0.CO;2-3 8726354

[142] Hoffman M Pizzo SV Weinberg JB. Alpha 2 Macroglobulin-Proteinase Complexes Stimulate Prostaglandin E2 Synthesis by Peritoneal Macrophages. Agents Actions (1988) 25:360–7. doi: 10.1007/BF01965043 2464277

[143] Uhing RJ Martenson CH Rubenstein DS Hollenbach PW Pizzo SV. The Exposure of Murine Macrophages to Alpha 2-Macroglobulin 'Fast' Forms Results in the Rapid Secretion of Eicosanoids. Biochim Biophys Acta (1991) 1093:115–20. doi: 10.1016/0167-4889(91)90111-a 1713784

[144] Misra UK Pizzo SV. Induction of Cyclooxygenase-2 Synthesis by Ligation of the Macrophage Alpha(2)-Macroglobulin Signalling Receptor. Cell Signal (2001) 13:801–8. doi: 10.1016/s0898-6568(01)00202-9 11583915

[145] Caceres LC Bonacci GR Sanchez MC Chiabrando GA. Activated Alpha(2) Macroglobulin Induces Matrix Metalloproteinase 9 Expression by Low-Density Lipoprotein Receptor-Related Protein 1 Through MAPK-ERK1/2 and NF-kappaB Activation in Macrophage-Derived Cell Lines. J Cell Biochem (2010) 111:607–17. doi: 10.1002/jcb.22737 20568116

[146] Roche PA Hoffman MR Pizzo SV. Effect of Interferon-Gamma and Human Alpha 2-Macroglobulin on Peritoneal Macrophage Morphology and Ia Antigen Expression. Biochim Biophys Acta (1990) 1051:166–73. doi: 10.1016/0167-4889(90)90189-k 1690028

[147] Ferrer DG Dato VA Jaldin-Fincati JR Lorenc VE Sanchez MC Chiabrando GA. Activated Alpha-2-Macroglobulin Induces Mesenchymal Cellular Migration of Raw264.7 Cells Through Low-Density Lipoprotein Receptor-Related Protein 1. J Cell Biochem (2017) 118:1810–8. doi: 10.1002/jcb.25857 28012205

[148] Misra UK Sharma T Pizzo SV. Ligation of Cell Surface-Associated Glucose-Regulated Protein 78 by Receptor-Recognized Forms of Alpha 2-Macroglobulin: Activation of P21-Activated Protein Kinase-2-Dependent Signaling in Murine Peritoneal Macrophages. J Immunol (2005) 175:2525–33. doi: 10.4049/jimmunol.175.4.2525 16081825

[149] Osada T Noro N Kuroda Y Ikai A. Murine T Cell Proliferation can be Specifically Augmented by Macrophages Fed With Specific Antigen: Alpha-2-Macroglobulin Conjugate. Biochem Biophys Res Commun (1987) 146:26–31. doi: 10.1016/0006-291x(87)90685-1 2440432

[150] Chu CT Oury TD Enghild JJ Pizzo SV. Adjuvant-Free *In Vivo* Targeting. Antigen Delivery by Alpha 2-Macroglobulin Enhances Antibody Formation. J Immunol (1994) 152:1538–45. 7509826

[151] Osada T Noro N Kuroda Y Ikai A. Antibodies Against Viral Proteins can be Produced Effectively in Response to the Increased Uptake of Alpha 2-Macroglobulin: Viral Protein Conjugate by Macrophages. Biochem Biophys Res Commun (1988) 150:883–9. doi: 10.1016/0006-291x(88)90475-5 2449204

[152] Morrot A Strickland DK Higuchi Mde L Reis M Pedrosa R Scharfstein J. Human T Cell Responses Against the Major Cysteine Proteinase (Cruzipain) of *Trypanosoma Cruzi*: Role of the Multifunctional Alpha 2-Macroglobulin Receptor in Antigen Presentation by Monocytes. Int Immunol (1997) 9:825–34. doi: 10.1093/intimm/9.6.825 9199965

[153] Binder RJ Han DK Srivastava PK. CD91: A Receptor for Heat Shock Protein Gp96. Nat Immunol (2000) 1:151–5. doi: 10.1038/77835 11248808

[154] Binder RJ Karimeddini D Srivastava PK. Adjuvanticity of Alpha 2-Macroglobulin, an Independent Ligand for the Heat Shock Protein Receptor CD91. J Immunol (2001) 166:4968–72. doi: 10.4049/jimmunol.166.8.4968 11290775

[155] Borth W Teodorescu M. Inactivation of Human Interleukin-2 (IL-2) by Alpha 2-Macroglobulin-Trypsin Complexes. Immunology (1986) 57:367–71. PMC1453844 2420701

[156] Heumann D Vischer TL. Immunomodulation by Alpha 2-Macroglobulin and Alpha 2-Macroglobulin-Proteinase Complexes: The Effect on the Human T Lymphocyte Response. Eur J Immunol (1988) 18:755–60. doi: 10.1002/eji.1830180515 2454194

[157] Petersen CM Ejlersen E Moestrup SK Jensen PH Sand O Sottrup-Jensen L. Immunosuppressive Properties of Electrophoretically "Slow" and "Fast" Form Alpha 2-Macroglobulin. Effects on Cell-Mediated Cytotoxicity and (Allo-) Antigen-Induced T Cell Proliferation. J Immunol (1989) 142:629–35. 2463311

[158] Monaco G Lee B Xu W Mustafah S Hwang YY Carre C. RNA-Seq Signatures Normalized by mRNA Abundance Allow Absolute Deconvolution of Human Immune Cell Types. Cell Rep (2019) 26:1627–40 e7. doi: 10.1016/j.celrep.2019.01.041 30726743 PMC6367568

[159] de Boer JP Creasey AA Chang A Abbink JJ Roem D Eerenberg AJ. Alpha-2-Macroglobulin Functions as an Inhibitor of Fibrinolytic, Clotting, and Neutrophilic Proteinases in Sepsis: Studies Using a Baboon Model. Infect Immun (1993) 61:5035–43. doi: 10.1128/iai.61.12.5035-5043.1993 PMC281280 7693593

[160] Abbink JJ Nuijens JH Eerenberg AJ Huijbregts CC Strack van Schijndel RJ Thijs LG. Quantification of Functional and Inactivated Alpha 2-Macroglobulin in Sepsis. Thromb Haemost (1991) 65:32–9. doi: 10.1055/s-0038-1647450 1708920

[161] Khan MM Shibuya Y Nakagaki T Kambara T Yamamoto T. Alpha-2-Macroglobulin as the Major Defence in Acute Pseudomonal Septic Shock in the Guinea-Pig Model. Int J Exp Pathol (1994) 75:285–93. PMC2002234 7524612

[162] Hochepied T Van Leuven F Libert C. Mice Lacking Alpha(2)-Macroglobulin Show an Increased Host Defense Against Gram-Negative Bacterial Sepsis, But Are More Susceptible to Endotoxic Shock. Eur Cytokine Netw (2002) 13:86–91. 11956025

[163] Lashin HMS Nadkarni S Oggero S Jones HR Knight JC Hinds CJ. Microvesicle Subsets in Sepsis Due to Community Acquired Pneumonia Compared to Faecal Peritonitis. Shock (2018) 49:393–401. doi: 10.1097/SHK.0000000000000989 28930915

[164] Birkenmeier G Nicklisch S Pockelt C Mossie A Steger V Glaser C. Polymyxin B-Conjugated Alpha 2-Macroglobulin as an Adjunctive Therapy to Sepsis: Modes of Action and Impact on Lethality. J Pharmacol Exp Ther (2006) 318:762–71. doi: 10.1124/jpet.106.104265 16705081

[165] Shapiro SD. Proteolysis in the Lung. Eur Respir J Suppl (2003) 44:30s–2s. doi: 10.1183/09031936.03.00000903a 14582898

[166] Kurdowska AK Geiser TK Alden SM Dziadek BR Noble JM Nuckton TJ. Activity of Pulmonary Edema Fluid Interleukin-8 Bound to Alpha(2)-Macroglobulin in Patients With Acute Lung Injury. Am J Physiol Lung Cell Mol Physiol (2002) 282:L1092–8. doi: 10.1152/ajplung.00378.2001 11943675

[167] Flory E Vischer TL. Alpha 2-Macroglobulin as an Inclusion in Synovial Fluid Monocytes. Rheumatol Int (1981) 1(2):61–4. doi: 10.1007/BF00541154 6180465

[168] Ekerot L Ohlsson K. Immunoreactive Alpha 2-Macroglobulin in Rheumatoid Synovial Membrane. Scand J Plast Reconstr Surg (1982) 16:293–4. doi: 10.3109/02844318209026222 6188207

[169] Flory ED Clarris BJ Muirden KD. Deposits of Alpha 2M in the Rheumatoid Synovial Membrane. Ann Rheum Dis (1982) 41:520–6. doi: 10.1136/ard.41.5.520 PMC1001034 6181747

[170] Abbink JJ Kamp AM Nieuwenhuys EJ Nuijens JH Swaak AJ Hack CE. Predominant Role of Neutrophils in the Inactivation of Alpha 2-Macroglobulin in Arthritic Joints. Arthritis Rheum (1991) 34:1139–50. doi: 10.1002/art.1780340910 1718287

[171] Moore AR Appelboam A Kawabata K Da Silva JA D'Cruz D Gowland G. Destruction of Articular Cartilage by Alpha 2 Macroglobulin Elastase Complexes: Role in Rheumatoid Arthritis. Ann Rheum Dis (1999) 58:109–13. doi: 10.1136/ard.58.2.109 PMC1752824 10343526

[172] Zhang Y Wei X Browning S Scuderi G Hanna LS Wei L. Targeted Designed Variants of Alpha-2-Macroglobulin (A2M) Attenuate Cartilage Degeneration in a Rat Model of Osteoarthritis Induced by Anterior Cruciate Ligament Transection. Arthritis Res Ther (2017) 19:175. doi: 10.1186/s13075-017-1363-4 28743292 PMC5526282

[173] Li S Xiang C Wei X Sun X Li R Li P. Early Supplemental Alpha2-Macroglobulin Attenuates Cartilage and Bone Damage by Inhibiting Inflammation in Collagen II-Induced Arthritis Model. Int J Rheum Dis (2019) 22:654–65. doi: 10.1111/1756-185X.13457 PMC6465088 30609267

[174] Liu X Liu L Zhang H Shao Y Chen Z Feng X. MiR-146b Accelerates Osteoarthritis Progression by Targeting Alpha-2-Macroglobulin. Aging (Albany NY) (2019) 11:6014–28. doi: 10.18632/aging.102160 PMC6738400 31422941

[175] Miyoshi S Shinoda S. Inhibitory Effect of Alpha 2-Macroglobulin on *Vibrio Vulnificus* Protease. J Biochem (1989) 106:299–303. doi: 10.1093/oxfordjournals.jbchem.a122848 2478526

[176] Coutinho CM Cavalcanti GH van Leuven F Araujo-Jorge TC. Alpha-2-Macroglobulin Binds to the Surface of *Trypanosoma Cruzi* . Parasitol Res (1997) 83:144–50. doi: 10.1007/s004360050224 9039696

[177] Ramos AM Duschak VG Gerez de Burgos NM Barboza M Remedi MS Vides MA. *Trypanosoma Cruzi*: Cruzipain and Membrane-Bound Cysteine Proteinase Isoform(s) Interacts With Human Alpha(2)-Macroglobulin and Pregnancy Zone Protein. Exp Parasitol (2002) 100:121–30. doi: 10.1016/S0014-4894(02)00007-3 12054702

[178] De Souza EM Meuser-Batista M Batista DG Duarte BB Araujo-Jorge TC Soeiro MN. *Trypanosoma Cruzi*: Alpha-2-Macroglobulin Regulates Host Cell Apoptosis Induced by the Parasite Infection *In Vitro* . Exp Parasitol (2008) 118:331–7. doi: 10.1016/j.exppara.2007.09.004 18028912

[179] Araujo-Jorge TC Lage MJ Rivera MT Carlier Y Van Leuven F. *Trypanosoma Cruzi*: Enhanced Alpha-Macroglobulin Levels Correlate With the Resistance of BALB/cj Mice to Acute Infection. Parasitol Res (1992) 78:215–21. doi: 10.1007/BF00931729 1375380

[180] Waghabi MC Coutinho CM Soeiro MN Pereira MC Feige JJ Keramidas M. Increased *Trypanosoma Cruzi* Invasion and Heart Fibrosis Associated With High Transforming Growth Factor Beta Levels in Mice Deficient in Alpha(2)-Macroglobulin. Infect Immun (2002) 70:5115–23. doi: 10.1128/IAI.70.9.5115-5123.2002 PMC128220 12183561

[181] Toppel AW Rasmussen M Rohde M Medina E Chhatwal GS. Contribution of Protein G-Related Alpha2-Macroglobulin-Binding Protein to Bacterial Virulence in a Mouse Skin Model of Group A Streptococcal Infection. J Infect Dis (2003) 187:1694–703. doi: 10.1086/375029 12751026

[182] Nyberg P Rasmussen M Bjorck L. Alpha2-Macroglobulin-Proteinase Complexes Protect *Streptococcus Pyogenes* From Killing by the Antimicrobial Peptide LL-37. J Biol Chem (2004) 279:52820–3. doi: 10.1074/jbc.C400485200 15520011

[183] Chen CH Zhang XQ Lo CW Liu PF Liu YT Gallo RL. The Essentiality of Alpha-2-Macroglobulin in Human Salivary Innate Immunity Against New H1N1 Swine Origin Influenza A Virus. Proteomics (2010) 10:2396–401. doi: 10.1002/pmic.200900775 PMC2890046 20391540

[184] Schramm W Seitz R Gurtler L. COVID-19-Associated Coagulopathy-Hypothesis: Are Children Protected Due to Enhanced Thrombin Inhibition by Higher Alpha2 -Macroglobulin Macroglobulin (Alpha2-M)? J Thromb Haemost (2020) 18:2416–8. doi: 10.1111/jth.15013 PMC7404876 32663364

[185] Seitz R Gurtler L Schramm W. Thromboinflammation in COVID-19: Can Alpha2 -Macroglobulin Help to Control the Fire? J Thromb Haemost (2021) 19:351–4. doi: 10.1111/jth.15190 PMC7753444 33230947

[186] Metzemaekers M Cambier S Blanter M Vandooren J de Carvalho AC Malengier-Devlies B. Kinetics of Peripheral Blood Neutrophils in Severe Coronavirus Disease 2019. Clin Transl. Immunology (2021) 10:e1271. doi: 10.1002/cti2.1271 PMC8082714 33968405

[187] Vanheule V Metzemaekers M Janssens R Struyf S Proost P. How Post-Translational Modifications Influence the Biological Activity of Chemokines. Cytokine (2018) 109:29–51. doi: 10.1016/j.cyto.2018.02.026 29903573

[188] Vandevyver S Dejager L Vandenbroucke RE Libert C. An Acute Phase Protein Ready to Go Therapeutic for Sepsis. EMBO Mol Med (2014) 6:2–3. doi: 10.1002/emmm.201303524 24408964 PMC3936485

[189] Cianciolo GJ Enghild JJ Pizzo SV. Covalent Complexes of Antigen and Alpha(2)-Macroglobulin: Evidence for Dramatically-Increased Immunogenicity. Vaccine (2001) 20:554–62. doi: 10.1016/s0264-410x(01)00361-9 11672922

[190] Vandendriessche S Cambier S Proost P Marques PE. Complement Receptors and Their Role in Leukocyte Recruitment and Phagocytosis. Front Cell Dev Biol (2021) 9:624025. doi: 10.3389/fcell.2021.624025 33644062 PMC7905230

